# Linagliptin prevents left ventricular stiffening by reducing titin cleavage and hypophosphorylation

**DOI:** 10.1111/jcmm.16122

**Published:** 2020-12-09

**Authors:** Ilona Cuijpers, Anna‐Pia Papageorgiou, Paolo Carai, Melissa Herwig, Andreas Mügge, Thomas Klein, Nazha Hamdani, Elizabeth A. V. Jones, Stephane Heymans

**Affiliations:** ^1^ Center for Molecular and Vascular Biology KU Leuven Leuven Belgium; ^2^ Department of Cardiology CARIM School for Cardiovascular Diseases Maastricht University Medical Center Maastricht The Netherlands; ^3^ Molecular Cardiology and Experimental Cardiology Ruhr University Bochum Bochum Germany; ^4^ Department of Cardiology St. Josef‐Hospital Ruhr University Bochum Bochum Germany; ^5^ Institute of Physiology Ruhr University Bochum Bochum Germany; ^6^ Boehringer Ingelheim Pharma GmbH & Co. KG Biberach Germany; ^7^ Department of Clinical Pharmacology Ruhr University Bochum Bochum Germany; ^8^ Holland Heart House ICIN‐Netherlands Heart Institute Utrecht The Netherlands

**Keywords:** cardiomyocyte passive stiffness, left ventricular stiffening, linagliptin, metabolic syndrome, titin

## Abstract

The metabolic syndrome (MetS) is an escalating problem worldwide, causing left ventricular stiffening, an early characteristic of diastolic dysfunction for which no treatment exists. As diastolic dysfunction and stiffening in MetS patients are associated with increased circulating dipeptidyl peptidase‐4 (DPP‐4) levels, we investigated whether the clinically approved DPP‐4 inhibitor linagliptin reduces left ventricular stiffness in MetS‐induced cardiac disease. Sixteen‐week‐old obese ZSF1 rats, displaying the MetS and left ventricular stiffness, received linagliptin‐supplemented or placebo diet for four weeks. Linagliptin significantly reduced obesity, hyperlipidaemia, and hyperglycaemia and improved left ventricular relaxation. This improved relaxation was related to decreased cardiac fibrosis and cardiomyocyte passive stiffness (*F*_passive_). The reduced *F*_passive_ was the result of titin isoform switching from the stiff N2B to the more flexible N2BA and increased phosphorylation of total titin and specifically its N2Bus region (S4080 and S3391). Importantly, DPP‐4 directly cleaved titin in vitro, resulting in an increased *F*_passive_, which was prevented by simultaneous administration of linagliptin. In conclusion, linagliptin improves left ventricular stiffness in obese ZSF1 rats by preventing direct DPP4‐mediated titin cleavage, as well as by modulating both titin isoform levels and phosphorylation. Reducing left ventricular stiffness by administering linagliptin might prevent MetS‐induced early diastolic dysfunction in human.

## 1 INTRODUCTION

The metabolic syndrome (MetS)—a cluster of hyperglycaemia, insulin resistance, obesity, hyperlipidaemia, and hypertension– is one of the most progressively escalating public health problems affecting children, adolescents, and adults worldwide. The MetS may cause left ventricular stiffening, an early manifestation of diastolic dysfunction.^1^ Impaired cardiomyocyte relaxation is the first and main determinant of left ventricular stiffness and precedes interstitial fibrosis. However, therapies to prevent the progression of the MetS towards left ventricular stiffening are lacking.

Cardiac stiffening involves increased cardiomyocyte stiffness (*F*_passive_),^2^ mainly due to modifications of the giant spring titin, which forms a continuous filament network in the sarcomeres of striated myocytes. Cardiac titin consists of two isoforms: the short, stiff N2B and the longer, more flexible N2BA isoform. Extension of the elastic I‐band segment in both isoforms allows for passive cardiomyocyte relaxation in diastole. Cardiomyocyte stiffness and left ventricular relaxation are modulated by isoform switching and phosphorylation of titin.^3^

Diastolic dysfunction and left ventricular stiffening are positively associated with elevated circulating dipeptidyl peptidase‐4 (DPP‐4) levels in type 2 diabetes and obese patients.^4^, ^5^, ^6^ The DPP‐4 inhibitor linagliptin is clinically used as an anti‐diabetic drug, as it improves blood glucose regulation by preventing the degradation of active gut‐derived glucagon‐like peptide 1 (aGLP‐1) and glucose‐dependent insulinotropic peptide (aGIP).^7^ Still the cardiovascular protective properties of DPP‐4 inhibitors remain a matter of debate^8^ and its protective effects on left ventricular stiffness and diastolic dysfunction are unclear. The present study revealed that DPP‐4 inhibition reduces left ventricular stiffening in a rat model of combined type 2 diabetes, hypertension, and obesity, in part by modulating titin cleavage, isoform switching, and phosphorylation. Linagliptin might be a potential strategy for the prevention and treatment of metabolic risk‐induced cardiac diastolic dysfunction in human.

## 2 MATERIAL AND METHODS

The raw data that support the findings of this study are available from the corresponding author upon reasonable request. A more detailed description of methods and full unedited gels are available in the Supporting Information.

### 2.1 Experimental animals

Experiments were performed according to the European Directive on the Care and Use of Experimental Animals (2010/63/EU) and approved by the Animal Care and Use Committee of KU Leuven (Project 283/2014). 8‐week‐old male ZSF1 rats, including obese (n = 14) and lean control (n = 4) littermates, were obtained from Charles River Laboratories (#strain code 378 and 379, respectively). ZSF1 rats are developed by crossing rat strains with two different types of leptin receptor mutations (*lepr fa* and *lepr facp*), the lean female ZDF (+/*lepr fa*) and the lean male SHHF (*SHHF/Mcc*; +/*lepr facp*) rat. The obese ZSF1 rat offspring are homozygous for the leptin receptor mutation (*lepr fa/lepr facp*) and inherit the hypertensive gene (*SHHF/Mcc*), resulting in the development of left ventricular stiffness secondary to type 2 diabetes, obesity and hypertension.^9^ In contrast to obese ZSF1 rats, lean ZSF1 littermates are heterozygous for the leptin receptor mutation (*lepr fa/+* or *lepr facp/+*) but inherit the hypertensive gene (*SHHF/Mcc*). Lean ZSF1 rats therefore only develop hypertension, thereby serving as controls. Animals were housed and acclimated under a 12‐hour light‐dark cycle with access to water and chow diet ad libitum (#V1534‐000, Ssniff Spezialdiäten GmbH).^9^

At 16 weeks of age, 7 obese ZSF1 rats (Obese + Lina) were randomized for linagliptin‐supplemented (83 mg/kg; Boehringer Ingelheim GmbH) chow diet (#V1534‐000, Ssniff Spezialdiäten GmbH),^10^, ^11^, ^12^ while 7 obese ZSF1 rats (Obese) and 4 lean ZSF1 rats (Lean) received the non‐supplemented chow diet ad libitum for 4 weeks. 83 mg/kg linagliptin in the chow diet corresponds to an estimated daily intake of 5‐8 mg/kg/bodyweight.^10^, ^12^, ^13^ As such, the administered dose in obese ZSF1 rats (average weight 490 grams at 16 weeks) corresponded to an oral dose of 2.45‐3.92 mg/day. We did not include linagliptin‐treated lean ZSF1 rats as a control, since we aimed to test the beneficial effects of the anti‐diabetic drug linagliptin in a combined type 2 diabetes‐, obesity‐, and hypertension‐induced cardiac disease model.

At 20 weeks, fasting blood glucose levels were assessed by Glucomen LX plus (A. Menarini Diagnostics) after 16 hours fasting in metal grid cages. Complete 2D echocardiography (B‐mode, M‐mode, pulsed wave Doppler, and tissue Doppler) using inhaled isoflurane (Ecuphar; 5% for induction and 2% for maintenance) was performed in 20‐week‐old lean ZSF1 rats and placebo‐ and linagliptin‐treated obese ZSF1 rats. The day after, rats were euthanized with 50 mg/kg ketamine (Nimatek, Eurovet Animal Health BV) and 5 mg/kg xylazine (Xyl‐M^®^, VMD nv/sa) by an intraperitoneal injection. Anaesthesia depth was confirmed by a toe pinch. Blood samples were collected for biochemical plasma measurements. Rats were then perfused with 20 mL PBS and organs were collected.

### 2.2 Force measurement in isolated cardiomyocytes ex vivo

Cardiac tissue was incubated in relaxation buffer supplemented with 1% Triton X‐100 (Sigma‐Aldrich) overnight to remove membrane structures. Isolated cardiomyocytes of linagliptin‐ and placebo‐treated obese ZSF1 rats were attached between a force transducer and length monitor (1600A; Aurora Scientific), and cardiomyocyte *F*_passive_ was measured between 1.8 and 2.4 µm sarcomere length.^9^

### 2.3 Myocardial kinase activities

Kinase activity was assessed in myocardial homogenates. Calcium/calmodulin‐dependent protein kinase II (CAMKII) and protein kinase (PK) A, C, and G activity were measured using a Cyclex CAMKII (#CY‐1173, MBL International), non‐radioactive PKA, PKC (#ADI‐EKS‐390A and −420A, Enzo Life Sciences), and home‐made radiolabelled ATP PKG activity kit, respectively.^14^

### 2.4 Titin isoform levels and phosphorylation

To determine titin isoform protein levels, left ventricular samples were mechanically homogenized, heated, and separated on an agarose strengthened 1.8% SDS‐PAGE gel for titin separation combined with a second 10% SDS‐PAGE gel for separation of small proteins at 2 mA overnight for at least 12 hours.^15^ Proteins were transferred on a polyvinylidene difluoride (PVDF) membrane and stained with Coomassie Blue. Titin and myosin heavy chain (MHC) bands were analysed,^16^ and titin N2B and N2BA isoform levels were normalized to MHC protein levels. To determine the phosphorylation level of N2B, the most abundant rodent isoform, an anti‐phospho serine (Ser)/threonine antibody (Thr) (dilution 1:500; #PP2551, ECM Biosciences LLC) was used to assess N2B phosphorylation. Both titin isoform and phosphorylation levels were normalized to control obese ZSF1 rats.

To determine site‐specific titin phosphorylation, previously validated affinity‐purified phospho site‐specific anti‐titin antibodies (Eurogentec) against N2B unique sequence (N2Bus) sites, including S3991, S4043 and S4080, and the region rich in proline, glutamic acid, valine and lysine (PEVK), including S12742 and S12884, were used.^9^, ^15^, ^17^, ^18^ Signals obtained from phospho‐specific antibodies were normalized to signals obtained from PVDF stains referring to the transferred protein amount.

### 2.5 In vitro human titin experiments

Left ventricular tissue of five male non‐failing human donors (average age 40 years), who died due to a non‐cardiac related death, was collected in cardioplegic solution and stored in liquid nitrogen until use. All procedures were performed according to the Declaration of Helsinki. Informed consent was obtained from all the participants and experiments were approved by the local ethics committee (OKAR/1066/2008/OKAR). Biopsies were incubated overnight in relaxing solution supplemented with 1% Triton X‐100. Stripped tissue was incubated with (a) 300 ng/mL recombinant his‐tagged DPP‐4 (# 50718‐M08H; Sino Biological),^19^ (b) 300 ng/mL recombinant his‐tagged DPP‐4 and 100 nmol/L linagliptin (Boehringer Ingelheim GmbH) ^20^, ^21^ or (c) PBS/DMSO (control) for 30 minutes to assess *F*_passive_ in vitro.

To determine site‐specific titin phosphorylation, previously validated affinity‐purified phospho site‐specific anti‐titin antibodies (Eurogentec) against N2B unique sequence (N2Bus) and phospho‐sites at positions S4010 and S4099 were used. Signals obtained from phospho‐specific antibodies were normalized to signals obtained from PVDF stains referring to the transferred protein amount.

To assess titin cleavage in vitro, both intact non‐permeabilized and non‐intact permeabilized (without and with Triton x‐100 pre‐treatment, respectively) human isolated cardiomyocytes were incubated with (a) 300 ng/mL his‐tagged recombinant DPP‐4, (b) 100 nmol/L linagliptin, (c) 300 ng/mL his‐tagged DPP‐4 and 100 nmol/L linagliptin, (d) or PBS/DMSO (control) at 37°C for 2 hours. Samples were run on an agarose‐strengthened 1.8% SDS‐PAGE gel on top of a 10% acrylamide SDS‐PAGE gel at 2 mA overnight for at least 12 hours. Cleavages of total titin were visualized by Coomassie Blue staining. Segment‐specific titin cleavage was assessed by western blotting using custom‐made, affinity‐purified antibodies, including the anti‐N2Bus domain against mouse sequence QELLSKETLFP and anti‐PEVK domain against (cross‐species conserved) sequence KLRPGSGGEKPP (both Eurogentec).

### 2.6 Statistical analysis

Data are expressed as mean ± SEM. All analysis was performed using GraphPad Prism 7.0. Normal distribution of all continuous variables was tested using the Shapiro‐Wilk normality test. Normally distributed data were analysed using a two‐tailed unpaired Student t test, while non‐normally distributed data were analysed using a Mann‐Whitney *U* test with *P < .05, **P < .01, and ***P < .001. Bodyweight over time was assessed with a two‐way ANOVA with *P < .05, **P < .01 and ***P < .001. Post‐translational modifications in stimulated human cardiomyocytes in vitro were analysed using a one‐way ANOVA with Dunnett's multiple comparison post hoc test with *P < .05, **P < .01 and ***P < .001 comparing control, DPP‐4 + Lina, and Lina to DPP‐4.

## 3 RESULTS

### 3.1 The obese ZSF1 rat as a model for MetS‐induced cardiac disease

As published previously, obese ZSF1 rats were obese, hyperglycaemic, and hyperlipidaemic and showed increased plasma DPP‐4 levels compared to lean control ZSF1 at 20 weeks (Figure S1A‐F).^22^, ^23^ Obese ZSF1 rats showed signs of MetS‐induced diastolic dysfunction and left ventricular stiffening, as reflected by a significantly increased mitral valve deceleration time, E/deceleration time ratio and non‐flow time (NFT), and a slightly, although non‐significantly increased isovolumic relaxation time (IVRT), early mitral inflow peak velocity (E), and E/E’ ratio (*P* = .06, .05, and .09, respectively) compared to lean ZSF1 rats (Figure S1G‐H and Table S1). In addition to signs of diastolic dysfunction, obese ZSF1 rats showed signs of cardiac remodelling, including increased total, perivascular, and interstitial fibrosis and cardiomyocyte hypertrophy (Figure S2A‐E), as published before.^9^, ^22^ As such, the obese ZSF1 rats could be used as a model of MetS‐induced cardiac diastolic dysfunction and remodelling.

### 3.2 Linagliptin improves cardiac relaxation in cardiometabolic disease

**FIGURE 1:**
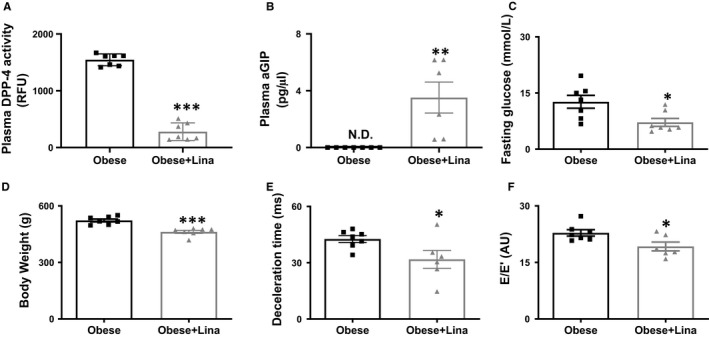
Linagliptin improves left ventricular relaxation in obese ZSF1 rats. Plasma DPP‐4 activity (A), aGIP levels (B), fasting glucose levels (C), bodyweight (D), mitral valve deceleration time (E) and E/E’ ratio (F) in 20‐wk‐old linagliptin‐ (Obese + Lina) and placebo‐treated obese (Obese) ZSF1 rats (n = 7 per group). aGIP, active glucose‐dependent insulinotropic peptide; DPP‐4, dipeptidyl peptidase‐4; E, early mitral inflow peak velocity; E’, early diastolic mitral annulus peak velocity; ND, not detected; RFU, relative fluorescence units. Data are expressed as mean ± SEM. Data were analysed using a two‐tailed unpaired Student t test, except D was analysed by a Mann‐Whitney *U* test. * Indicates *P* < .05, **P < .01 and ***P < .001

As diastolic dysfunction and stiffening in MetS patients are associated with increased circulating DPP‐4 levels, we investigated whether the clinically approved DPP‐4 inhibitor linagliptin reduces left ventricular stiffness in MetS‐induced cardiac disease in obese ZSF1 rats.^4^, ^5^, ^6^ Four weeks of linagliptin supplementation significantly reduced fasting glucose levels by reducing DPP‐4 activity, resulting in decreased breakdown of systemic aGIP and aGLP‐1 (Figure 1A‐C and Figure S3A). Furthermore, linagliptin‐treated rats had significantly decreased bodyweight, organ (liver, kidney, and spleen) weight‐to‐tibia length (TL) ratio, plasma triglycerides and non‐high‐density lipoprotein (HDL) cholesterol levels, while HDL cholesterol was increased (Figure 1D and Figure S3B‐E and Table S2). In addition to its well‐established anti‐diabetic, anti‐obesity, and anti‐hyperlipidaemic effects,^7^, ^24^ linagliptin is suggested to have beneficial cardiac effects.^25^ For example, Aroor et al showed that linagliptin improved diastolic function in insulin‐resistant male Zucker obese rats.^12^ Interestingly, mitral valve deceleration time and E/E’, the non‐invasive parameter for left ventricular stiffness, were significantly reduced in linagliptin‐treated obese ZSF1 rats (Figure 1E‐F and Table S3), indicating decreased left ventricular stiffness and improved relaxation. Paradoxically, IVRT was increased in linagliptin‐treated obese ZSF1 rats (Table S3), and this indicates that the development of diastolic dysfunction only could be partially prevented. Thus, linagliptin decreases cardiometabolic risk factors, including obesity, hyperglycaemia, and hyperlipidaemia, and improved left ventricular relaxation in obese ZSF rats.

### 3.3 Linagliptin has cardiac anti‐fibrotic effects in obese ZSF1 rats

**FIGURE 2:**
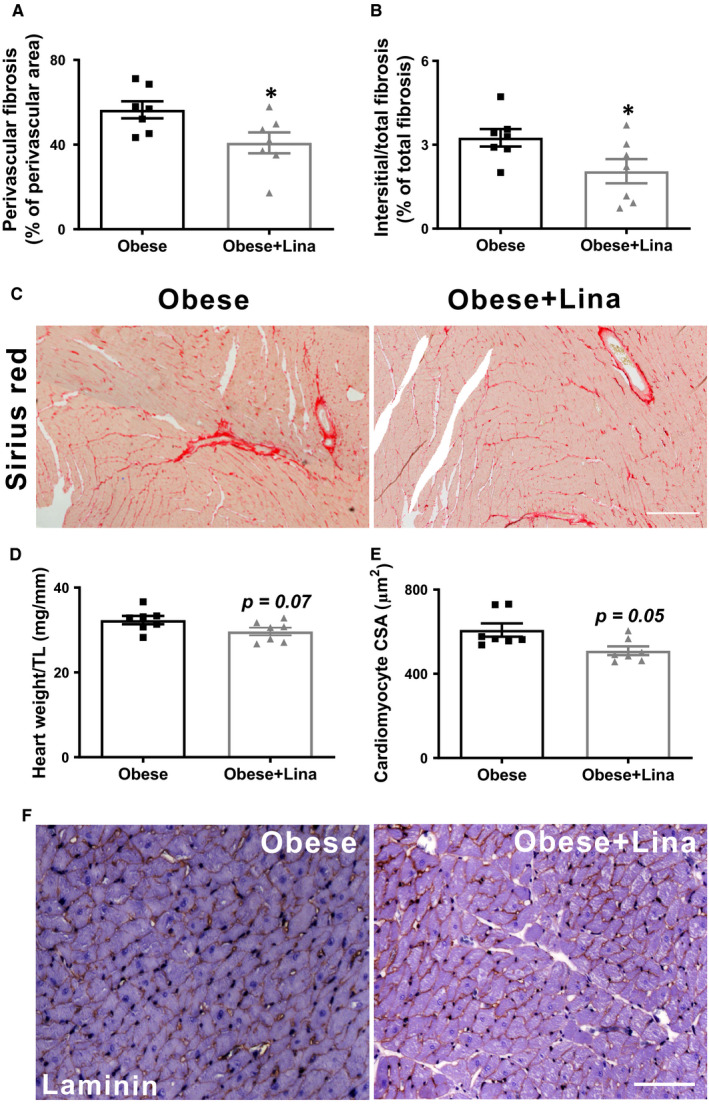
Linagliptin has anti‐fibrotic effects without changing cardiomyocyte hypertrophy in obese ZSF1 rats. A‐C, Quantification and representative images of perivascular and interstitial cardiac fibrosis of Sirius red‐stained sections in 20‐wk‐old linagliptin‐ (Obese + Lina) and placebo‐treated obese (Obese) ZSF1 rats (n = 7 per group; scale bar = 150 µm). Heart weight‐to‐tibia length (D), cardiomyocyte cross‐sectional area (E), and laminin‐stained cardiac sections (F; scale bar = 100 µm) in linagliptin‐ (Obese + Lina) and placebo‐treated obese (Obese) ZSF1 rats (both n = 7). CSA, cross‐sectional area; TL, tibia length. Data are expressed as mean ± SEM. A and B were analysed using a two‐tailed unpaired Student *t* test, and D and E were analysed by a Mann‐Whitney *U* test with *P < .05

Increased left ventricular stiffness may in part be determined by extracellular matrix‐mediated stiffness, caused by elevated cardiac fibrosis, while reductions in cardiac fibrosis are associated with a reduced left ventricular stiffness.^3^ Linagliptin significantly reduced total, perivascular, and interstitial cardiac fibrosis in obese ZSF1 rats (Figure 2A‐C and Figure S4A‐B). Cardiac transcript levels of genes involved in fibrosis, including *Col1a1*, *Col3a1* and *Timp1* were also significantly decreased after linagliptin treatment, while cardiac *Mmp2*, *Mmp9* and *Timp2* levels were not significantly different between linagliptin‐ and placebo‐treated obese ZSF1 rats (Figure S4C‐H). Linagliptin also mildly reduced heart weight/TL ratio and cardiomyocyte size, without reaching statistical significance (*P* = .07 and .05, respectively, Figure 2D‐F). This indicates both anti‐fibrotic and mild anti‐hypertrophic effects, in line with previous rodent studies.^26^, ^27^

### 3.4 Linagliptin reduces cardiomyocyte passive stiffness by isoform switching to the more flexible N2BA and post‐translational modifications of titin

**FIGURE 3:**
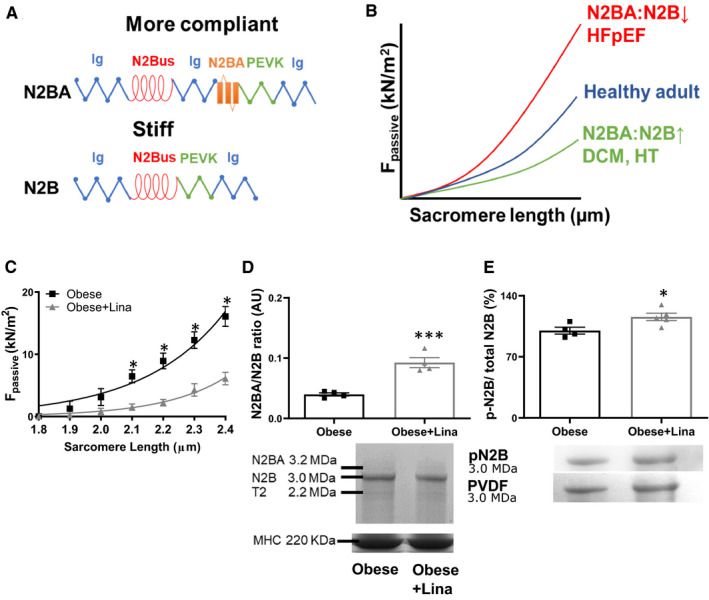
Linagliptin induces isoform switching and total titin phosphorylation in obese ZSF1 rats. A, Structure composition of the extensible I‐band of the more compliant N2BA and stiffer N2B titin isoform. Blue, Ig domain; red, N2Bus domain; green, PEVK domain; orange, N2BA domain. B, Consequence of isotype switching on cardiomyocyte *F*_passive_. Titin isoform switching from the N2BA to the N2B isoform (reduced N2BA:N2B ratio) increases *F*_passive_, as observed HFpEF, while the inverse reduces *F*_passive_, as shown in dilated DCM and HT. C, Cardiomyocyte passive stiffness in isolated cardiomyocytes of linagliptin‐ (Obese + Lina) and placebo‐treated obese (Obese) ZSF1 rats at 20 wk (n = 3 different left ventricular tissues measuring at least 12 cardiomyocytes from each left ventricular tissue per condition). N2BA/N2B titin isoform ratio and representative Coomassie Blue stained‐PVDF membranes containing bands of N2BA, N2B and titin‐2 (T2; known degradation product), and loading control MHC (D) and total N2B titin phosphorylation and representative Coomassie Blue stained‐PVDF membranes (E) in linagliptin‐ (Obese + Lina) and placebo‐treated obese (Obese) ZSF1 rats at 20 wk (n = 4‐7 per group). DCM, dilated cardiomyopathy; *F*_passive_, passive stiffness; HFpEF, heart failure with preserved ejection fraction; HT, hypothyroidism; Ig, immunoglobulin; MHC, myosin heavy chain; N2Bus, N2B unique sequence. Data are expressed as mean ± SEM. All data were analysed using a two‐tailed unpaired Student *t* test with *P < .05 and ***P < .001

The primary determinant of left ventricular stiffness in early diastolic dysfunction is an increased cardiomyocyte *F*_passive_, mainly determined by modifications of the giant spring protein titin.^3^ In the adult heart, titin has two predominant isoforms: the longer and more compliant N2BA isoform and the shorter and stiffer N2B isoform (Figure 3A). The adult human myocardium contains roughly similar amounts of the more compliant N2BA and stiffer N2B isoforms, while N2B is the predominant isoform in the rodent myocardium.^28^, ^29^ Titin isoform switching from the N2BA to the N2B isoform (reduced N2BA:N2B ratio) increases *F*_passive_, as observed in diastolic dysfunction in heart failure with preserved ejection fraction (HFpEF), while the inverse reduces *F*_passive_, as shown in dilated cardiomyopathy (DCM) and hypothyroidism (HT) (Figure 3B).^30^ Linagliptin significantly reduced *F*_passive_ in isolated cardiomyocytes from obese ZSF1 rats (Figure 3C), along with an increased N2BA/N2B ratio (Figure 3D), indicating titin isoform switching from the stiffer N2B to the more flexible N2BA isoform (Figure S5A‐B).

**FIGURE 4:**
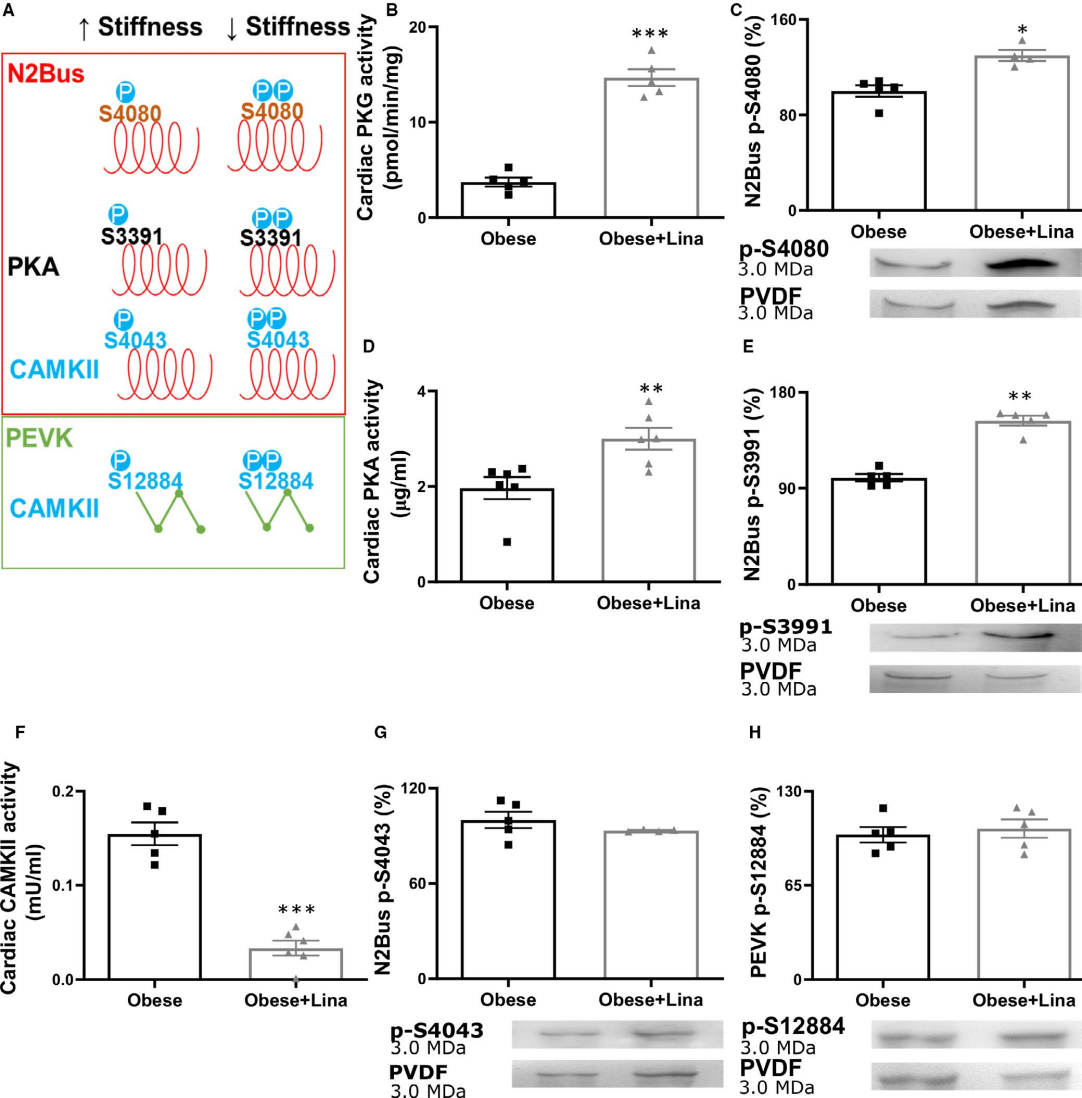
Linagliptin prevents N2Bus segment hypophosphorylation in obese ZSF1 rats. A, Site‐specific phosphorylation by protein kinases alters cardiomyocyte passive stiffness. Cardiac PKG activity (B), PKG‐dependent N2Bus S4080 phosphorylation and representative Coomassie Blue stained‐PVDF membranes (C), cardiac PKA activity (D), PKA‐mediated N2Bus S3991 phosphorylation and representative Coomassie Blue stained‐PVDF membranes (E), cardiac CAMKII activity (F) and CAMKII‐mediated N2Bus S4043 phosphorylation and representative Coomassie Blue stained‐PVDF membranes (G), and PEVK S12884 phosphorylation and representative Coomassie Blue stained‐PVDF membranes (H) in 20 wk‐old linagliptin‐ (Obese + Lina) and placebo‐treated obese (Obese) ZSF1 rats (both n = 5‐7). CAMKII, calcium/calmodulin‐dependent protein kinase II; N2Bus, N2B unique sequence; P, phosphorylation; PKA, protein kinase A; PKG, protein kinase G. Data are expressed as mean ± SEM. B, F, G and H were analysed using a two‐tailed unpaired Student t test, C‐E were analysed by a Mann‐Whitney *U* test with *P < .05, **P < .01 and ***P < .001

In addition to isoform switching, phosphorylation of titin is also an important modulator of *F*_passive_. Hypophosphorylation of the N2B titin isoform occurs in patients and animals with diastolic dysfunction.^9^, ^31^ Linagliptin increased titin N2B isoform phosphorylation in obese ZSF1 rats (Figure 3E). The cardiac‐specific isoform N2B can be phosphorylated at the N2Bus and/ PEVK region within the I‐band by various protein kinases, such as PKA, PKCα, PKG, and CAMKII. PKG phosphorylates the N2Bus segment at S4080 (conserved; called S4099 in humans) and hypophosphorylation is associated with increased *F*_passive_ in patients with dilated and hypertrophic cardiomyopathy and canine HFpEF (Figure 4A).^17^, ^32^ Linagliptin significantly increased cardiac PKG activity and thereby increased N2Bus S4080 phosphorylation in obese ZSF1 rats, implying a potential mechanism for the reduced *F*_passive_ (Figure 4B‐C). PKA phosphorylates titin at the N2Bus segment at S3991 (conserved; referred to S4010 in humans) and hypophosphorylation is associated with increased *F*_passive_ (Figure 4A).^9^ Linagliptin also significantly increased cardiac PKA activity and PKA‐dependent phosphorylation of the N2Bus S3391, which is associated with a reduced *F*_passive_ (Figure 4D‐E). In addition, titin is phosphorylated by CAMKII at the N2Bus region on S4043 (conserved; called S4062 in humans) and hypophosphorylation is associated with an increased *F*_passive_ (Figure 4A).^33^ PKCα can also phosphorylate titin at PEVK S12742 (conserved; named S11878 in humans) and hyperphosphorylation is associated with an increased *F*_passive_.^34^ Lastly, both CAMKII and PKCα phosphorylate titin at the PEVK region on S12884 (conserved; S12022 in humans) and CAMKII‐mediated hypophosphorylation is associated with an increased *F*_passive_ (Figure 4A).^9^, ^33^ Linagliptin significantly reduced cardiac CAMKII and PKC activity in obese ZSF1 rats, while CAMKII‐dependent phosphorylation at N2Bus S4043 and PEVK S12884 and PKC‐dependent PEVK S12742 phosphorylation were not affected (Figure 4F‐H and Figure S5C‐D).

Thus, linagliptin reduces *F*_passive_ in obese ZSF1 rats by inducing isoform switching from the stiff N2B to the more flexible N2BA isoform and post‐translational modifications of the giant spring protein titin, including increased phosphorylation of total titin, and PKG‐ and PKA‐dependent site‐specific phosphorylation of N2Bus S4080 and S3391, respectively.

### 3.5 Linagliptin prevents DPP‐4‐mediated titin cleavage and titin hypophosphorylation in human cardiomyocytes

**FIGURE 5:**
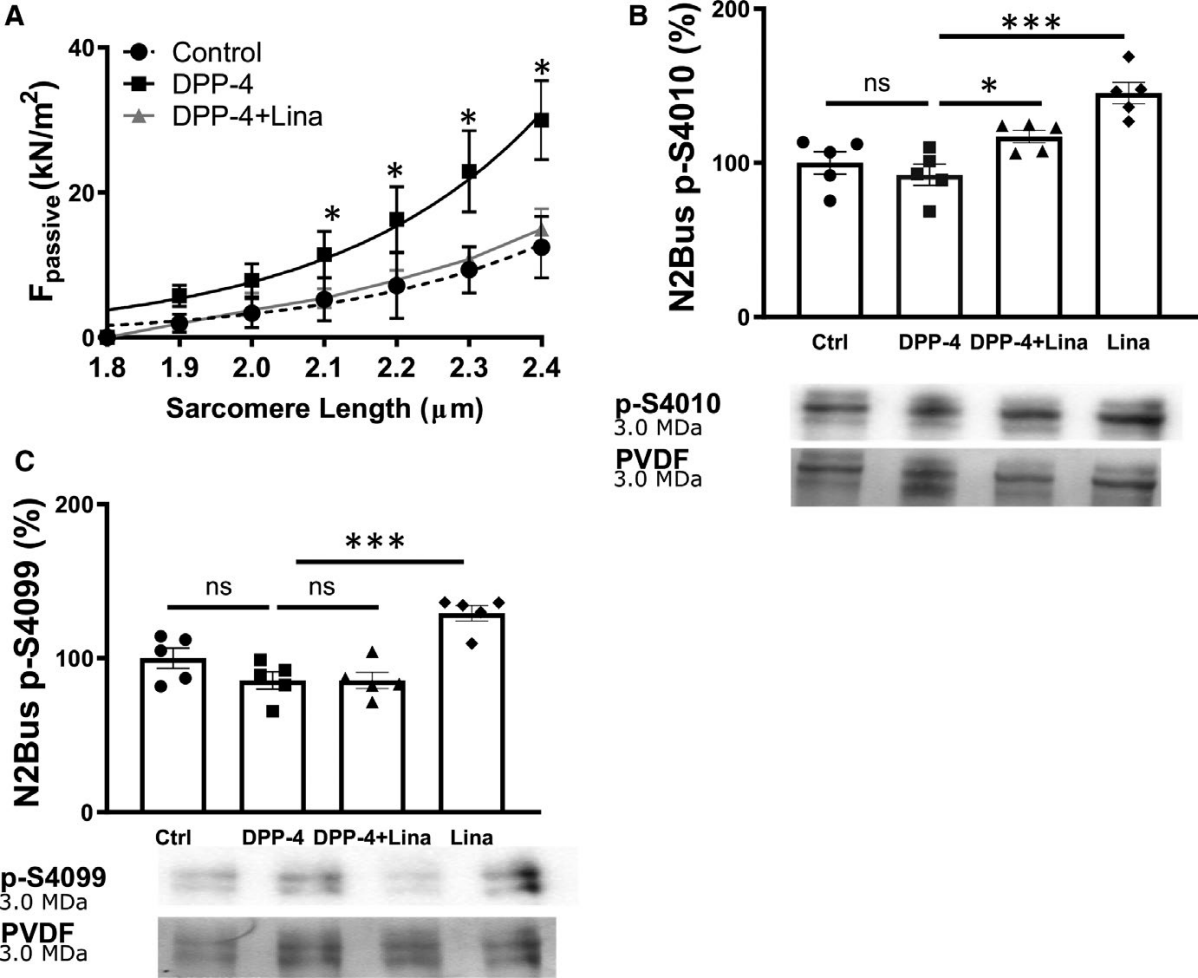
Linagliptin decreases *F*_passsive_ by increasing titin phosphorylation at N2Bus S4010 in human cardiomyocytes in vitro. A, Cardiomyocyte passive stiffness in human cardiomyocytes treated with 300 ng/mL DPP‐4, 100 nmol/L linagliptin and 300 ng/mL DPP‐4, or control (DMSO/PBS) for 30 min (n = 3 different left ventricular tissues measuring at least 12 cardiomyocytes from each left ventricular tissue per condition). B, PKA‐mediated N2Bus S4010 phosphorylation and representative Coomassie Blue stained‐PVDF membranes and (C) PKG‐dependent N2Bus S4099 phosphorylation in human cardiomyocytes (n = 5 different left ventricular tissues) treated with vehicle control (DMSO/PBS), 300 ng/mL DPP‐4, 300 ng/mL DPP‐4 and 100 nmol/L linagliptin, or 100 nmol/L linagliptin for 2 h. *F*_passive_, passive stiffness; N2Bus, N2B unique sequence. Panel A was analysed using a two‐tailed unpaired Student t test with *P < .05. Panel B and C were analysed using a one‐way ANOVA with Dunnett's multiple comparison post hoc test with *P < .05 and ***P < .001 comparing control, DPP‐4 + Lina and Lina to DPP‐4

In addition to linagliptin's indirect cardioprotective effects by decreasing cardiometabolic risk factors, DPP‐4 inhibitors may also have direct local effect in the cardiomyocyte. In line with previous findings, we observed that DPP‐4, one of the main targets of linagliptin, is expressed in the heart (Figure S6A).^35^ While DPP‐4 was predominantly expressed by coronary capillaries and immune cells, some cardiomyocytes also express DPP‐4 within the cytosol (Figure S6A; black arrows), as published previously.^35^, ^36^ Furthermore, gliptins (saxagliptin and sitagliptin) are internalized by cardiomyocytes and localize to the cytosol.^37^, ^38^ As such, we first investigated whether linagliptin could directly affect cardiomyocyte *F*_passive_ in vitro in human cardiomyocytes in the absence of cardiometabolic risk factors. We incubated isolated human cardiomyocytes with either DPP‐4, linagliptin and DPP‐4, or vehicle control. Incubation with DPP‐4 resulted in an increased *F*_passive_ compared to control, while addition of linagliptin completely prevented the DPP‐4‐induced increase in *F*_passive_ similar to our in vivo data in obese ZSF1 rats (Figure 5A).

We next assessed whether linagliptin treatment affects PKA‐ and PKG‐dependent post‐translational modifications in human cardiomyocytes in vitro similar to those observed in vivo. In line with our in vivo data, simultaneous treatment of human cardiomyocytes with DPP‐4 and linagliptin resulted in increased phosphorylation of N2Bus S4010 (S3991 in rats) compared to DPP‐4‐treated human cardiomyocytes (Figure 5B). In contrast to our in vivo data, we could not detect any differences in PKG‐mediated phosphorylation of N2Bus S4099 (S4080 in rats) between human cardiomyocytes treated with both linagliptin and DPP‐4 or DPP‐4‐treated human cardiomyocytes (Figure 5C).

**FIGURE 6:**
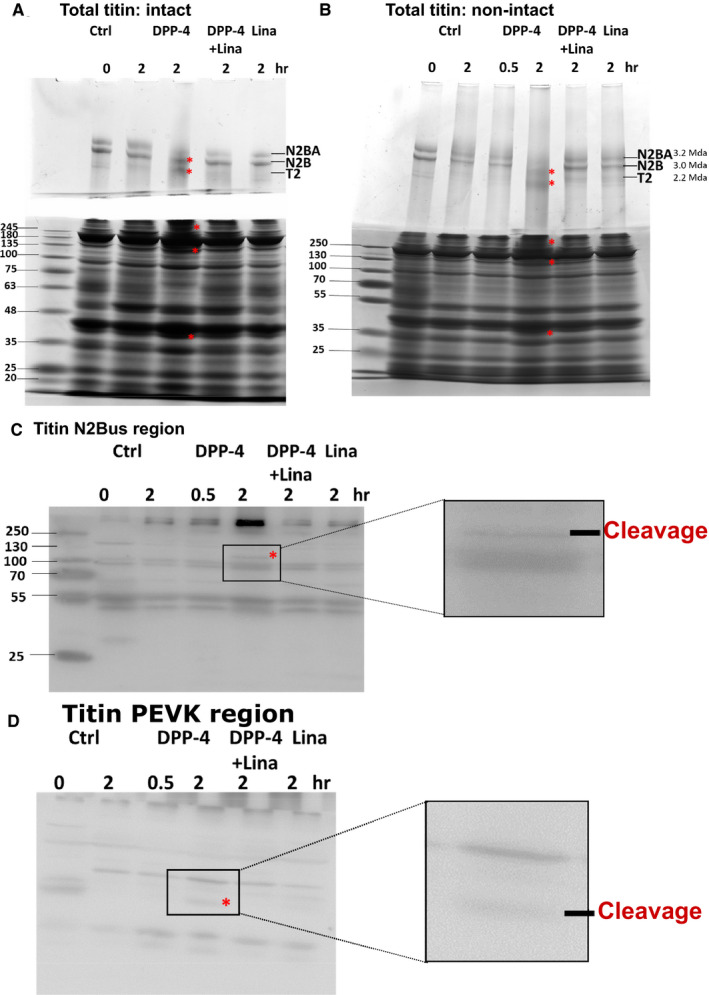
Linagliptin prevents DPP‐4‐mediated titin cleavage in human cardiomyocytes in vitro. A‐B, Western blots of total titin, containing the two isoforms N2BA and N2B and the known degradation product titin‐2 (T2) of human intact non‐permeabilized (A) and non‐intact permeabilized (B) human cardiomyocytes exposed to control PBS/DMSO (Ctrl) for 0 or 2 h (lane 1 and 2, respectively), 300 ng/mL DPP‐4 for 30 min and 2 h (lane 3 and 4, respectively), 300 ng/mL DPP‐4 and 100 nmol/L linagliptin for 2 h (lane 5) or 100 nmol/L linagliptin alone for 2 h (lane 6) in vitro (n = 3 different left ventricular tissues). Titin cleavage is indicated by red asterisks. Western blots of the specific N2Bus region (C; cross‐species conserved sequence QELLSKETLFP) and PEVK region (D; cross‐species conserved sequence KLRPGSGGEKPP) of human cardiomyocytes exposed to control PBS/DMSO (Ctrl) for 0 or 2 h (lane 1 and 2, respectively), 300 ng/mL DPP‐4 for 30 min and 2 h (lane 3 and 4, respectively), 300 ng/mL DPP‐4 and 100 nmol/L linagliptin for 2 h (lane 5) or 100 nmol/L linagliptin alone for 2 h (lane 6) (n = 3 different left ventricular tissues). Titin cleavage is indicated by red asterisks. The framed area represents the location for the zoomed images presented on the right side. Ctrl, control; DPP‐4, dipeptidyl peptidase‐4; *F*_passive_, passive stiffness; Lina, linagliptin; N2Bus, N2B unique sequence. Data are expressed as mean ± SEM

Importantly, treating intact non‐permeabilized and permeabilized (incubated with 1% Triton) cardiomyocytes with DPP‐4 induced titin cleavage in vitro, while simultaneous linagliptin administration with DDP‐4 prevented this (Figure 6A‐B; cleavage products indicated with red asterisks). Visualization of titin protein levels at the full N2Bus and anti‐PEVK domain of human N2B titin form revealed cleavage products in permeabilized cardiomyocytes after administration of DPP‐4 in vitro, while simultaneous linagliptin and DPP‐4 treatment prevented titin cleavage at both the full N2Bus and PEVK region (Figure 6C‐D and Figure S6B‐C; cleavage products indicated with red asterisks). Thus, linagliptin may also prevent elevated passive stiffness in part by directly inhibiting DPP‐4‐mediated titin cleavage.

This indicates that linagliptin reduces cardiomyocyte *F*_passive_ in human cardiomyocytes in vitro by preventing titin cleavage and increasing PKA‐dependent site‐specific phosphorylation of N2Bus S4010 (S3391 in rat).

## 4 DISCUSSION

**FIGURE 7:**
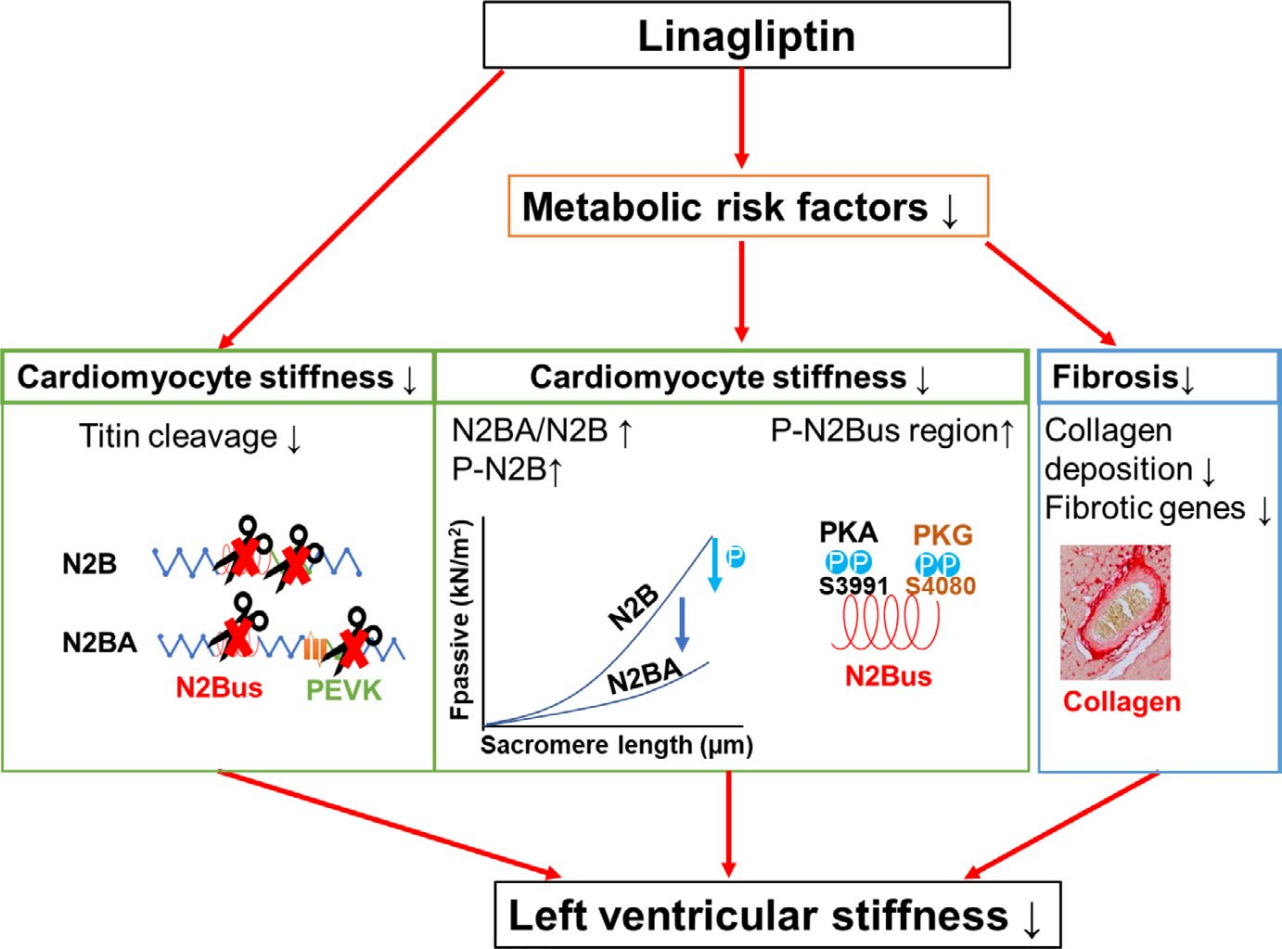
Proposed mechanism underlying the reduced left ventricular stiffening in metabolic syndrome‐induced cardiac disease after linagliptin administration. *F*_passive_, passive stiffness; N2Bus, N2B unique sequence; PEVK, proline (P), glutamic acid (E), valine (V) and lysine (K)

Here, linagliptin, a commonly used oral drug for the treatment of type 2 diabetes, significantly decreased left ventricular passive stiffness in a rat model of MetS‐induced left ventricular dysfunction (Figure 7). The improvement of left ventricular passive stiffness upon linagliptin treatment was mainly caused by decreasing the first and main determinant of left ventricular stiffness, called cardiomyocyte *F*_passive_. This reduced cardiomyocyte *F*_passive_ was the result of the prevention of DPP‐4 mediated titin cleavage in vitro, as well as isotype switching from the stiff N2B isoform towards the more flexible N2BA titin isoform and increased total and site‐specific phosphorylation of N2Bus titin at S4080 and S3391 in vivo. In addition, cardiac fibrosis, another determinant of left ventricular stiffening, which develops after cardiomyocyte stiffening, was decreased by linagliptin, as described before in individual models of either type 2 diabetes, obesity, or hypertension.^27^, ^39^, ^40^

In addition to linagliptin's cardioprotective effects mediated by reducing metabolic risk factors (eg hyperglycaemia and hyperlipidaemia), we found that linagliptin directly prevented DPP‐4‐mediated cleavage of titin, specifically in the N2Bus and PEVK regions, resulting in decreased cardiomyocyte *F*_passive_ in vitro. Breakdown of titin may predispose cardiomyocytes to diastolic dysfunction, myofilament instability, and cell death.^41^ Our findings are in line with previous studies, where titin cleavage had been associated with cardiac stiffening and failure in humans^42^ and targeted deletion of the N2Bus or PEVK region of the N2B titin isoform led to increased *F*_passive_ and diastolic dysfunction in mice.^43^, ^44^ Thus, part of the beneficial effect of linagliptin treatment on *F*_passive_ and diastolic dysfunction may be explained by preventing DPP4‐mediated titin cleavage.

The beneficial effect of linagliptin on cardiomyocyte stiffness in vivo was also accompanied by isoform switching from the stiff N2B isoform to the more compliant N2BA titin isoform in obese ZSF1 rats, most probably as a result of its positive effect on the components of the MetS, especially type 2 diabetes.^45^ For example, insulin administration has been shown to affect N2B titin isoform levels and titin post‐translational modification in rat cardiomyocytes in vitro.^45^, ^46^ Interestingly, linagliptin is the first anti‐cardiometabolic risk therapy that induces isotype switching to the more flexible N2BA isoform, while other therapies, such as metformin^47^ and exercise training,^48^ did not show any changes in titin isoforms and another DPP‐4 inhibitor sitagliptin even induced switching to the stiffer N2B isoform.^49^ However, as linagliptin functions as an insulin sensitizer, we can not rule out that the isoform switching observed in obese ZSF1 rats is the result of indirect alteration of the local insulin signalling in the cardiomyocytes.

Linagliptin also increased total and site‐specific phosphorylation of titin at N2Bus S3391 and S4080 in obese ZSF1 rats. In contrast to isoform switching, post‐translational modifications of titin, including phosphorylation by kinases (eg PKG and PKA), provide a faster mechanism to change cardiomyocyte *F*_passive_. Hypophosphorylation of the total N2B isoform contributes to increased *F*_passive_ in human and animal hearts of HFpEF,^9^, ^50^ while site‐specific hypophosphorylation of N2Bus S3991 and S4080 occurs in rodent HFpEF^9^, ^47^ and type 2 diabetes models.^46^ In line with our results, diabetic mice treated with sitagliptin had increased total titin phosphorylation, which were suggested to be partly mediated by stimulatory effects on the myocardial PKG pathway.^49^ However, sitagliptin's effects on site‐specific phosphorylation and cleavage of titin were not assessed in this study. Furthermore, as patients suffering from the MetS often represent with renal impairments, linagliptin is preferred as it is uniquely primarily eliminated via a non‐renal route,^51^ while sitagliptin is predominantly excreted via the kidneys and requires dose adjustments in patients with renal dysfunction.^52^ Altogether, linagliptin reduces cardiomyocyte stiffness by increasing titin total and site‐specific phosphorylation at N2Bus S4080 and S3991.

In short, linagliptin decreased left ventricular stiffness in MetS‐induced cardiovascular disease. Linagliptin reduced cardiomyocyte *F*_passive_ by modifying titin's isoform levels and total and site‐specific phosphorylation indirectly via its beneficial effect on the metabolic risk factors. Importantly, linagliptin also directly reduced cardiomyocyte *F*_passive_ by preventing DPP‐4‐mediated titin cleavage. Whereas in human clinical studies DPP‐4 inhibition does not prevent heart failure hospitalizations, it remains unknown whether it may prevent the development of early diastolic dysfunction and the progression towards HFpEF. Reducing left ventricular stiffness by administering linagliptin might therefore be considered as a potential strategy for the prevention and treatment of metabolic risk‐induced cardiac diastolic dysfunction in human.

## CONFLICT OF INTEREST

TK is an employee of Boehringer Ingelheim International GmbH. All the authors declare that they have no conflict of interest.

## AUTHOR CONTRIBUTION

**Ilona Cuijpers:** Conceptualization (lead); Data curation (lead); Formal analysis (lead); Funding acquisition (supporting); Investigation (lead); Methodology (lead); Project administration (lead); Supervision (lead); Validation (lead); Visualization (lead); Writing‐original draft (lead); Writing‐review & editing (lead). **Anna‐Pia Papageorgiou:** Conceptualization (lead); Data curation (supporting); Formal analysis (lead); Funding acquisition (lead); Investigation (lead); Methodology (lead); Project administration (lead); Supervision (supporting); Writing‐review & editing (equal). **Paolo Carai:** Investigation (supporting); Project administration (supporting); Writing‐review & editing (equal). **Melissa Herwig:** Investigation (supporting); Writing‐review & editing (equal). **Andreas Mügge:** Resources (supporting); Writing‐review & editing (equal). **Thomas Klein:** Resources (lead); Writing‐review & editing (equal). **Nazha Hamdani:** Conceptualization (supporting); Formal analysis (supporting); Investigation (supporting); Writing‐review & editing (equal). **Elizabeth Jones:** Conceptualization (supporting); Funding acquisition (lead); Supervision (lead); Writing‐review & editing (supporting). **Stephane Heymans:** Conceptualization (supporting); Funding acquisition (lead); Supervision (lead); Writing‐review & editing (supporting).

## Supporting information

Supplementary Material

## Data Availability

The data that support the findings of this study are available from the corresponding author upon reasonable request.
