# Supplementary material for: Linagliptin prevents left ventricular stiffening by reducing titin cleavage and hypophosphorylation
Source: J Cell Mol Med. 2020 Dec 9;25(2):729–41. doi: 10.1111/jcmm.16122 (PMC7812306; doi:10.1111/jcmm.16122)
Supplement: Supplementary file 1 — Supplementary Material [file JCMM-25-729-s001.docx]

**Linagliptin prevents left ventricular stiffening by reducing titin cleavage and hypophosphorylation**

Ilona Cuijpers^1,2*^, Anna-Pia Papageorgiou^1,2*^, Paolo Carai^1^, Melissa Herwig^3,4,5^, Andreas Mügge^3,4^, Thomas Klein^6^, Nazha Hamdani^3,4,5,7#^, Elizabeth A. V. Jones^1,2#^, Stephane Heymans^1,2,8#^

*,^#^ Authors contributed equally

^1^ Center for Molecular and Vascular Biology, KU Leuven, Leuven, Belgium

^2^ Department of Cardiology, Maastricht University Medical Center, CARIM School for Cardiovascular Diseases, Maastricht, Netherlands

^3^ Molecular Cardiology and Experimental Cardiology, Ruhr University Bochum, Bochum, Germany

^4^ Department of Cardiology, St. Josef-Hospital, Ruhr University Bochum, Bochum, Germany

^5^ Institute of Physiology, Ruhr University Bochum, Bochum, Germany

^6^ Boehringer Ingelheim Pharma GmbH & Co. KG, Biberach, Germany

^7^ Department of Clinical Pharmacology, Ruhr University Bochum, Bochum, Germany

^8^ ICIN-Netherlands Heart Institute, Holland Heart House, Utrecht, Netherlands

**Short title:** *Linagliptin blocks cardiac stiffening*

Category: Original Article

Total word count: 3853

**Corresponding author:**

Prof. Stephane Heymans, MD

Department of Cardiology, Maastricht University Medical Center

CARIM School for Cardiovascular Diseases,

Universiteitssingel 50, PO BOX 5800,

6229 ER Maastricht, Netherlands

Tel: 0031433877097

Fax: 0031433871055

# [s.heymans@maastrichtuniversity.nl](mailto:s.heymans@maastrichtuniversity.nl)

# **1. Supporting Information Material and Methods**

## **1.1 Transthoracic echocardiography**

Rats were anesthetized by inhaling 5% inhaled isoflurane (Ecuphar, Breda, Netherlands) for induction followed by 2% inhaled isoflurane for maintenance. Complete transthoracic echocardiography was performed using a MS 250 transducer (13-24 MHz) connected to a Vevo 2100 echocardiograph (Visual Sonics, Toronto, Canada). Heart rate (HR), end-systolic and -diastolic volume (ESV and EDV, respectively), and left ventricular dimensions, including anterior wall thickness (AW), left ventricular internal diameter (LVID), and posterior wall thickness (PW), were assessed on the parasternal short-axis B-mode. Cardiac output (CO), stroke volume (SV), ejection fraction (EF), fractional shortening (FS), and left ventricular volume (LVV) were calculated based on parasternal short-axis B-mode recordings. Left ventricular filling was assessed by pulsed wave Doppler trans-mitral flow velocity tracings, including peak early (E) and late (A) wave velocities, mitral valve deceleration time, no-flow time (NFT), aortic ejection time (AET), isovolumic contraction and relaxation time (IVCT and IVRT, respectively), and myocardial performance index (MPI), just above the tip of the mitral leaflets. Myocardial early (E’) and late (A’) diastolic peak velocity were measured by tissue Doppler imaging at the lateral mitral annulus. E/A, E/deceleration time, and E/E’ ratios were calculated. At least three stable cardiac cycles were averaged for all measurements.

## **1.2 Animal euthanasia**

At 20 weeks of age, rats were anesthetized with 50 mg/kg ketamine (Nimatek, Eurovet Animal Health BV, Bladel, Netherlands) and 5 mg/kg xylazine (Xyl-M®, V.M.D. nv/sa, Arendonk, Belgium) dissolved in 0.9% NaCl by an intraperitoneal injection. Anaesthesia depth was confirmed by a toe pinch. Blood samples were collected in EDTA-coated vacutainer tubes (BD Biosciences, Erembodegem, Belgium) from the posterior vena cava and plasma was used for biochemical measurements. Rats were perfused with 20 ml PBS by inserting a butterfly needle in the left ventricle, while making a cut in the right atrium. Rats were euthanized by excision of the heart and organs were weighted and collected for histological and molecular analyses at 20 weeks. Organ weights were normalized by tibia length (TL), expressed as grams per millimetre.

## **1.3 Biochemical plasma measurements**

Plasma dipeptidyl peptidase-4 (DPP-4), active gut-derived glucagon-like peptide (aGLP-1), and glucose-dependent insulinotropic peptide (aGIP) levels were measured by Boehringer Ingelheim International GmbH (Biberbach, Germany)^1, 2^. Plasma triglyceride, total cholesterol, high-density lipoprotein (HDL), and non-HDL cholesterol were measured by the clinical laboratory (UZ Leuven, Leuven, Belgium) after centrifugation of the plasma for 10 minutes at 1500 x g.

## **1.4 RNA isolation and quantitative RT-PCR**

Total RNA was isolated from snap-frozen cardiac tissue using the miRVana kit (Thermofisher, Merelbeke, Belgium). For quantitative RT-PCR, 1 µg of total RNA was reversed transcribed in cDNA using the miScript II RT kit (Qiagen, Netherlands) and transcript levels in 5 ng cDNA were quantified using SYBR Green PCR Master Mix (Thermofisher, Merelbeke, Belgium) and Applied Biosystems QuantStudio 3 (Thermofisher, Merelbeke, Belgium). Primers were designed to span exon-exon junctions using Primer3 software and synthesized by integrated DNA technologies (Leuven, Belgium). Relative gene expression levels were calculated using the 2^-ΔΔCt^ method.

| **Gene** | **Forward primer (5’-3’)** | **Reverse primer (5’-3’)** |
| --- | --- | --- |
| ***Col1a1*** | aggagagagtgccaactcca | gtagggagcagcaagag |
| ***Col3a1*** | ggcaatcctgatcttcctga | gcctgatccatgtaggcaat |
| ***Mmp2*** | ggaracaggrgrgccaaggt | gcagtgtggtggaaaactga |
| ***Mmp9*** | agcctgtggttggtcagaag | ataaaagggccggtaaggtg |
| ***Timp1*** | tcccttgcaaactggagagt | gtcatcgagaccccaaggta |
| ***Timp2*** | ccctccaaccagtgtttcat | ggggaagagagaggaaggaa |
| ***Gapdh*** | gtggacctcatggcctacat | tgtgagggagatgctcagtg |
|  |  |  |

## **1.5 Histology**

Cardiac tissue was fixed in 1% paraformaldehyde at 4 °C for 24 hours and imbedded in paraffin (Leica TP1020; Leica Biosystems, Machelen, Belgium). Cardiac sections (4 μm) were stained with Picro Sirius Red (fibrosis) and rabbit anti-Laminin A antibodies (1/400; #L9393, Sigma-Aldrich, Overijse, Belgium). To assess the location of DPP-4 in the heart, PFA sections of obese ZSF1 rats were stained with DPP-4 (1/50; ab114033, Abcam, Cambridge, UK). Images were acquired using an Axiovert 200M microscope (Zeiss, Oberkochen, Germany) and analysis was performed using Image J software. The amount of total cardiac fibrosis was quantified as the percentage Sirius red positive area per total cardiac area. For perivascular fibrosis, close to the outer vessel wall border a circle was drawn. The vessel was cut out of the mosaic image. The amount of perivascular fibrosis (excluding staining artefacts) was assessed in the perivascular area. The perivascular area, including the vessel area and collagen (excluding the vessel lumen), was measured. Perivascular fibrosis and area were measured for all the vessels with a diameter of >10 µm. The percentage of perivascular fibrosis was calculated by dividing the total amount of perivascular fibrosis by the total perivascular area. Interstitial fibrosis was calculated as total fibrosis minus perivascular fibrosis. Interstitial fibrosis was expressed as a percentage of total fibrosis. Cardiomyocyte hypertrophy was assessed by calculating the myocyte cross-sectional area (CSA) based on the inner circumference of at least 240 myocytes per laminin-stained section.

## **1.6 Titin isoform levels and phosphorylation**

To determine titin N2B and N2BA isoform protein levels, left ventricular samples were mechanically homogenized in a modified Laemmli buffer containing 50 mM Tris-HCl, pH 6.8, 8 M urea, 2 M thiourea, 3% SDS (w/v), 0.03 % ServaBlue (w/v), 10% (v/v) glycerol, and 75 mM DTT. After heating at 96 °C for 3 minutes and centrifugation, samples were separated on an agarose-strengthened 1.8% SDS-PAGE gel for titin separation and combined with a second 10% SDS-PAGE gel for the separation of small proteins at 2 mA overnight for at least 12 hours. Proteins were transferred on a polyvinylidene difluoride (PVDF) membrane using a semi-dry technique (25V, 1.3A for 7 minutes). PVDF membranes were stained with Coomassie Blue (0.0075% in methanol). Titin and myosin heavy chain (MHC) bands were analysed by densitometry as previously described^3^. Titin isoform levels were normalized to MHC protein levels. To determine the phosphorylation level of total titin, anti-phospho serine (Ser)/threonine (Thr) (dilution 1:500; #P0444801-2, ECM Biosciences LLC, Versailles, KY) was used to assess N2B phosphorylation. Both titin isoform and phosphorylation levels were normalized to placebo-treated obese ZSF1 rats.

To determine site-specific titin phosphorylation, samples were homogenized, heated, separated by 1.8% SDS-PAGE combined with a 10% SDS-PAGE gel and transferred on a PVDF membrane, as described above. PVDF membranes were stained with Coomassie Blue, washed with TBST, and blocked with 3% BSA in TBST for 1 hour at room temperature. Then blots were incubated overnight at 4°C with the phospho site-specific anti-titin antibodies, which were previously validated custom-made by Eurogentec (Belgium) against positions in N2B unique sequence (N2Bus) and a region rich in proline, glutamate, valine and lysine amino acids (PEVK) domains of Mus musculus titin according to UniProtKB identifier A2ASS6. The listed antibodies below have previously been verified^4-7^:

- anti-phospho-N2Bus (Ser3991 in rats) against EEGKS(PO3H2)LSFPLA (dilution 1:500);
- anti-phospho-N2Bus (Ser4043) against QELLS(PO3H2)KETLFP (dilution 1:100) and anti-mouse-N2Bus-titin against mouse sequence QELLSKETLFP (dilution 1:500)
- anti-phospho-N2Bus (Ser4080 in rats) against LFS(PO3H2)EWLRNI (dilution 1:500);
- anti-phospho-PEVK (Ser12742) against EVVLKS(PO3H2)VLRK (dilution 1:100);
- anti-phospho-PEVK (Ser12884) against KLRPGS(PO3H2)GGEKPP (dilution 1:500) and anti-PEVK-domain against (cross-species conserved) sequence KLRPGSGGEKPP (1:100).

The amino acid sequences of rat are identical to the amino acid sequences of mouse^5, 8^. The amino acid sequences of rat titin at Ser3991 and Ser4080 are identical to the amino acid sequences of mouse, and refer to human titin at Ser4010 and Ser4099. After washing with TTBS, primary antibody binding was visualized using secondary horseradish peroxidase(HRP)-labelled, goat anti-rabbit antibody (1/10,000; #P044801-2, Dako Cytomation, Denmark) and enhanced chemiluminescence (ECL Western blotting detection; Amersham Biosciences, France). Stainings were visualized using the LAS-4000 Image Reader and analysed with Multi Gauge V3.2 software (both from FUJIFILM Corp, Japan). Coomassie PVDF stainings were saved for comparison of protein load. Finally, signals obtained from phospho-specific antibodies were normalized to signals obtained from PVDF stains referring to the entire protein amount transferred.

# **2. Supplementary Tables**

**Table S1: Echocardiography in lean and obese ZSF1 rats at 20 weeks**

| Parameter | Lean  (n=4) | Obese  (n=7) | P-value |
| --- | --- | --- | --- |
| HR (bpm) | 344 ± 12.4 | 291 ± 7.34 | **0.0034** |
| SV (µl) | 307 ± 15.4 | 408 ± 37.0 | 0.0797 |
| CO (ml/min) | 105 ± 5.39 | 120 ± 12.9 | 0.4391 |
| AWd (mm) | 1.99 ± 0.10 | 1.95 ± 0.09 | 0.8155 |
| AWs (mm) | 2.76 ± 0.09 | 2.90 ± 0.09 | 0.3259 |
| LVIDd (mm) | 7.90 ± 0.09 | 8.77 ± 0.36 | 0.1090 |
| LVIDs (mm) | 5.70 ± 0.14 | 5.89 ± 0.29 | 0.6624 |
| PWd (mm) | 2.48 ± 0.09 | 2.06 ± 0.11 | **0.0301** |
| PWs (mm) | 3.16 ± 0.14 | 2.90 ± 0.10 | 0.1670 |
| EDV (µl) | 563 ± 3.10 | 680 ± 53.8 | 0.1430 |
| ESV (µl) | 257 ± 17.4 | 273 ± 30.5 | 0.7257 |
| LVV (µl) | 429 ± 33.0 | 419 ± 26.9 | 0.8159 |
| EF (%) | 54.4 ± 2.93 | 60.0 ± 2.81 | 0.2352 |
| FS (%) | 27.8 ± 1.31 | 32.9 ± 1.86 | 0.0953 |
| E (mm/s) | 877 ± 76.4 | 1070 ± 50.0 | 0.0539 |
| A (mm/s) | 471 ± 80.4 | 513 ± 33.2 | 0.5862 |
| E/A (AU) | 1.97 ± 0.24 | 2.13 ± 0.16 | 0.5729 |
| Deceleration time(ms) | 24.2 ± 3.03 | 42.6 ± 1.79 | **0.0003** |
| E/deceleration time | 37.3 ± 3.92 | 25.4 ± 1.53 | **0.0078** |
| IVRT (ms) | 28.2 ± 1.47 | 32.4 ± 1.22 | 0.0647 |
| IVCT (ms) | 17.2 ± 1.55 | 20.0 ± 1.40 | 0.2475 |
| AET (ms) | 64.6 ± 3.60 | 74.4 ± 2.79 | 0.0622 |
| NFT (ms) | 110 ± 2.23 | 127 ± 4.61 | **0.0298** |
| E’ (mm/s) | 44.6 ± 2.31 | 46.9 ± 1.41 | 0.3865 |
| A’ (mm/s) | 34.2 ± 2.72 | 31.0 ± 1.58 | 0.2972 |
| E/E’ (AU) | 19.7 ± 1.67 | 22.8 ± 0.84 | 0.0946 |
| MPI (AU) | 0.71 ± 0.08 | 0.70 ± 0.01 | 0.8546 |

Data are presented as mean ± SEM. A, arterial or “late” mitral inflow peak velocity; A’, late diastolic mitral annulus peak velocity; AET, aortic ejection time; AW, anterior wall; CO, cardiac output; d, diastole; E, early mitral inflow peak velocity; E’, early diastolic mitral annulus peak velocity; EDV, end-diastolic volume; EF, ejection fraction; ESV, end-systolic volume; FS, fractional shortening; HR, heart rate; IVCT, isovolumic contraction time; IVRT, isovolumic relaxation time; LVID, left ventricular internal diameter; LVV, left ventricular volume; MPI, myocardial performance index; NFT, non-flow time; PW, posterior wall; s, systole; SV, stroke volume.

**Table S2: Plasma lipid levels in linagliptin- and placebo-treated obese ZSF1 rats**

|  | Obese  (n=7) | Obese + Lina  (n=7) | P-values |
| --- | --- | --- | --- |
| Triglycerides (mg/dl) | 471 ± 50.7 | 227 ± 23.4 | **0.0009** |
| Cholesterol (mg/dl) | 104 ± 6.32 | 91.7 ± 10.3 | 0.3452 |
| HDL cholesterol (mg/dl) | 19.4 ± 1.49 | 34.0 ± 4.53^**^ | **0.0100** |
| Non-HDL cholesterol (mg/dl) | 84.1 ± 6.16 | 57.9 ± 8.13^*^ | **0.0242** |

Values are presented as mean ± SEM. HDL indicates high-density lipoprotein.

**Table S3: Echocardiography in placebo-and linagliptin-treated obese** **ZSF1 rats at 20 weeks**

| Parameter | Obese  (n=7) | Obese + Lina  (n=7) | P-value |
| --- | --- | --- | --- |
| HR (bpm) | 291 ± 7.34 | 283 ± 5.93 | 0.3753 |
| SV (µl) | 408 ± 37.0 | 420 ± 30.9 | 0.8051 |
| CO (ml/min) | 120 ± 12.9 | 118 ± 8.16 | 0.9242 |
| AWd (mm) | 1.95 ± 0.09 | 1.83 ± 0.10 | 0.3822 |
| AWs (mm) | 2.90 ± 0.09 | 2.71 ± 0.19 | 0.3830 |
| LVIDd (mm) | 8.77 ± 0.36 | 9.13 ± 0.35 | 0.4866 |
| LVIDs (mm) | 5.89 ± 0.29 | 6.46 ± 0.41 | 0.2858 |
| PWd (mm) | 2.06 ± 0.11 | 1.99 ± 0.09 | 0.6234 |
| PWs (mm) | 2.90 ± 0.10 | 2.64 ± 0.16 | 0.3176 |
| EDV (µl) | 680 ± 53.8 | 755 ± 61.6 | 0.3777 |
| ESV (µl) | 273 ± 30.5 | 335 ± 39.9 | 0.2352 |
| LVV (µl) | 419 ± 26.9 | 392 ± 31.1 | 0.5309 |
| EF (%) | 60.0 ± 2.81 | 56.3 ± 2.87 | 0.3793 |
| FS (%) | 32.9 ± 1.86 | 29.6 ± 2.46 | 0.3119 |
| E (mm/s) | 1070 ± 50.0 | 947 ± 32.2 | 0.1375 |
| A (mm/s) | 513 ± 33.2 | 501 ± 47.1 | 0.8438 |
| E/A (AU) | 2.13 ± 0.16 | 1.96 ± 0.16 | 0.4454 |
| Deceleration time (ms) | 42.6 ± 1.79 | 31.8 ± 4.77 | **0.0457** |
| E/deceleration time | 25.4 ± 1.53 | 27.2 ± 6.25 | 0.5347 |
| IVRT (ms) | 32.4 ± 1.22 | 36.9 ± 0.81 | **0.0135** |
| IVCT (ms) | 20.0 ± 1.40 | 16.5 ± 0.70 | 0.0831 |
| AET (ms) | 74.4 ± 2.79 | 78.9 ± 3.86 | 0.3553 |
| NFT (ms) | 127 ± 4.61 | 135 ± 3.63 | 0.2123 |
| E’ (mm/s) | 46.9 ± 1.41 | 50.3 ± 3.71 | 0.3817 |
| A’ (mm/s) | 31.0 ± 1.58 | 36.4 ± 3.23 | 0.1472 |
| E/E’ (AU) | 22.8 ± 0.84 | 19.2 ± 1.19 | **0.0279** |
| MPI (AU) | 0.70 ± 0.01 | 0.72 ± 0.06 | 0.7041 |

Data are presented as mean ± SEM. A, arterial or “late” mitral inflow peak velocity; A’, late diastolic mitral annulus peak velocity; AET, aortic ejection time; AW, anterior wall; CO, cardiac output; d, diastole; E, early mitral inflow peak velocity; E’, early diastolic mitral annulus peak velocity; EDV, end-diastolic volume; EF, ejection fraction; ESV, end-systolic volume; FS, fractional shortening; HR, heart rate; IVCT, isovolumic contraction time; IVRT, isovolumic relaxation time; Lina, linagliptin; LVID, left ventricular internal diameter; LVV, left ventricular volume; MPI, myocardial performance index; NFT, non-flow time; PW, posterior wall; s, systole; SV, stroke volume.

# **3. Supplementary Figures**


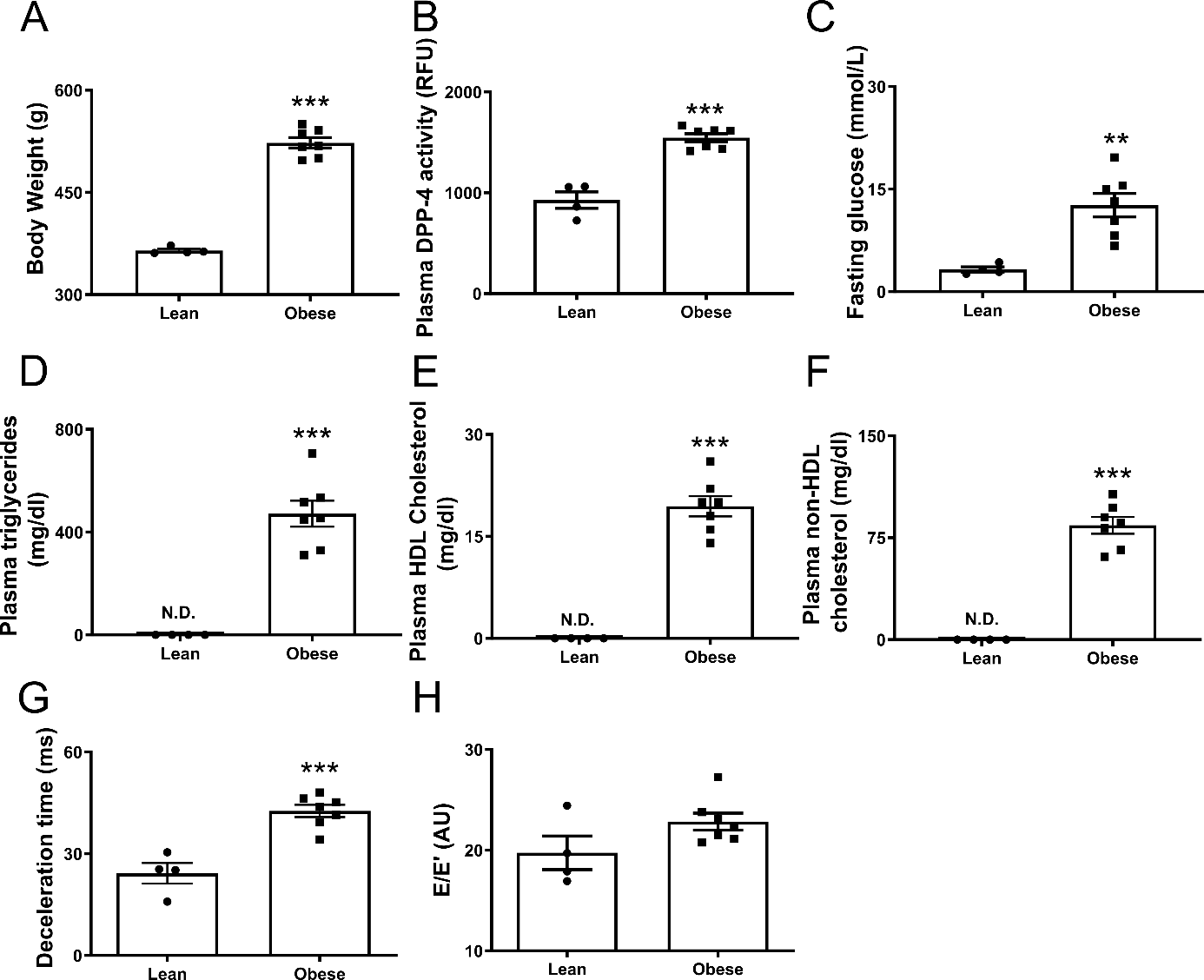


**Figure S1- Obese ZSF1 rats show obesity, hyperglycaemia, hyperlipidaemia, and signs of diastolic dysfunction.** Body weight (A), plasma DPP-4 activity (B), fasting glucose levels (C) plasma triglyceride (D), HDL cholesterol (E), and non-HDL cholesterol levels (F), mitral valve deceleration time (G), and E/E’ ratio (H) in 20-week-old lean (Lean; n=4) and obese (Obese; n=7) ZSF1 rats. DPP-4, dipeptidyl peptidase-4; E, early mitral inflow peak velocity; E’, early diastolic mitral annulus peak velocity; HDL, high-density lipoprotein; N.D., not detectable triglyceride levels (<9.0 mg/dl) and (non-)HDL-cholesterol (<12 mg/dl); RFU, relative fluorescence units. Data are expressed as mean ± SEM. All data were analysed using a two-tailed unpaired Student t-test with **P<0.01 and ***P<0.001.


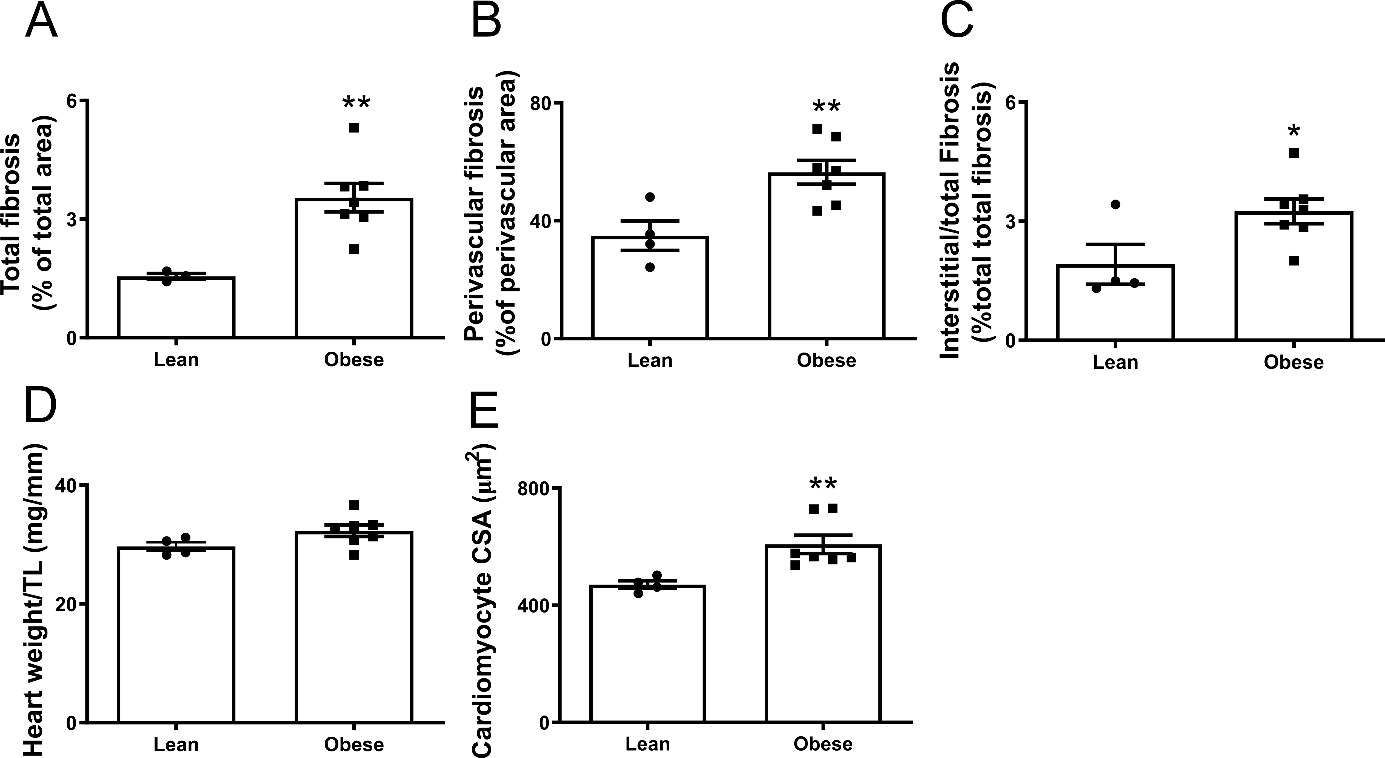


**Figure S2- Obese ZSF1 rats show cardiac fibrosis and hypertrophy.** Cardiac total (A), perivascular (B), and interstitial fibrosis (C), heart weight-to-tibia length (D), and cardiomyocyte cross-sectional area (E) in 20-week-old lean (Lean; n=4) and obese (Obese; n=7) ZSF1 rats. CSA, cross-sectional area; TL, tibia length. All data are expressed as mean ± SEM. A-D were analysed using a two-tailed unpaired Student t-test and E was analysed by a Mann-Whitney U test with *P<0.05, and **P<0.01.

**
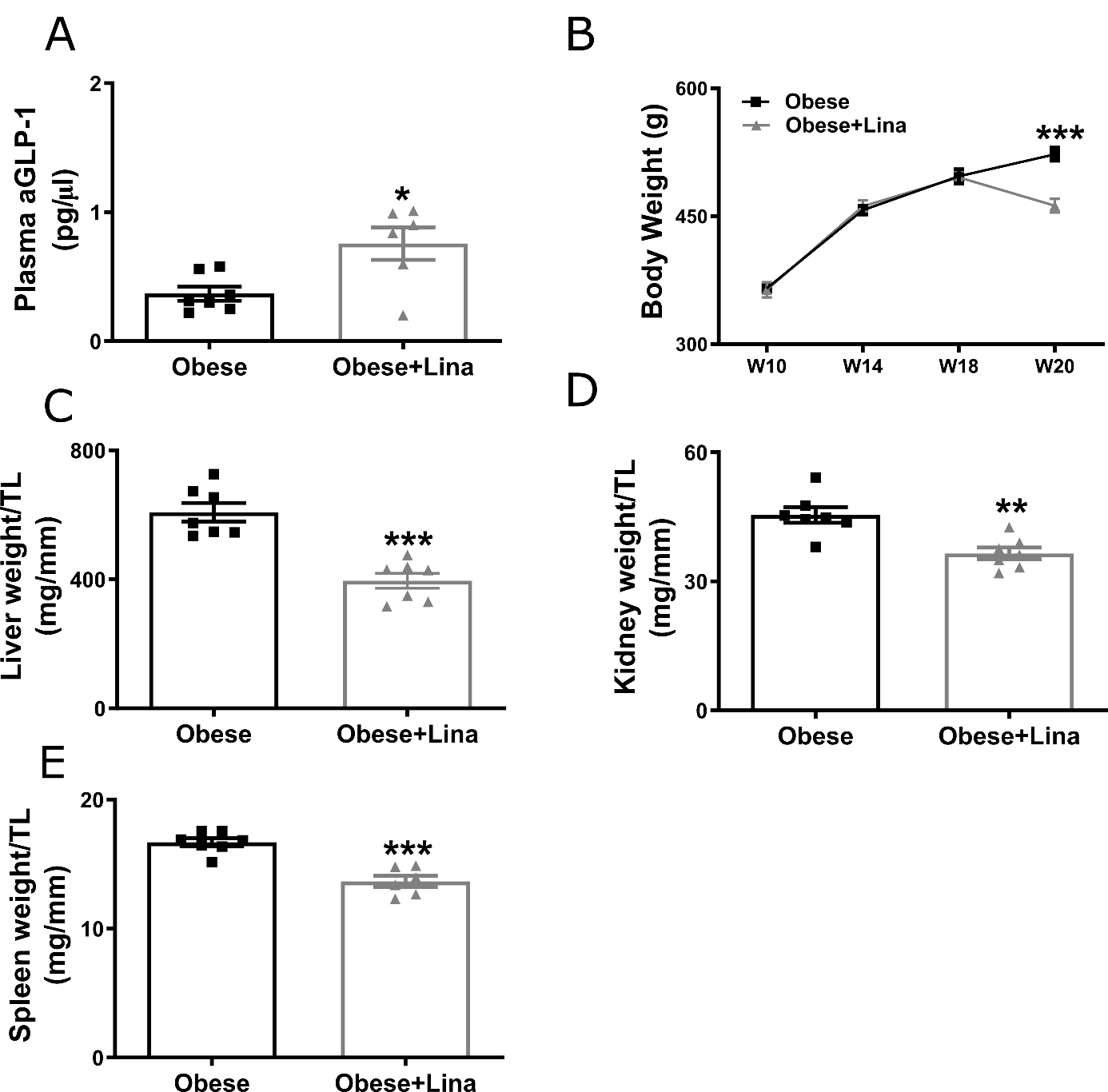
**

**Figure S3- Linagliptin increases plasma aGLP-1, while decreasing body and organ weights in obese ZSF1 rats.** Plasma aGLP-1 (A), body weight over time (B), and liver (C), kidney (D), and spleen (E) weight to tibia length in linagliptin- (Obese+Lina) and placebo-treated obese (Obese) ZSF1 rats (both =7). aGLP-1, active glucagon-like peptide 1; TL, tibia length. Data are expressed as mean ± SEM. A, C-E were analysed using a two-tailed unpaired Student t-test and B was analysed by a two-way ANOVA. * Indicates P<0.05, **P<0.01, and ***P<0.001.


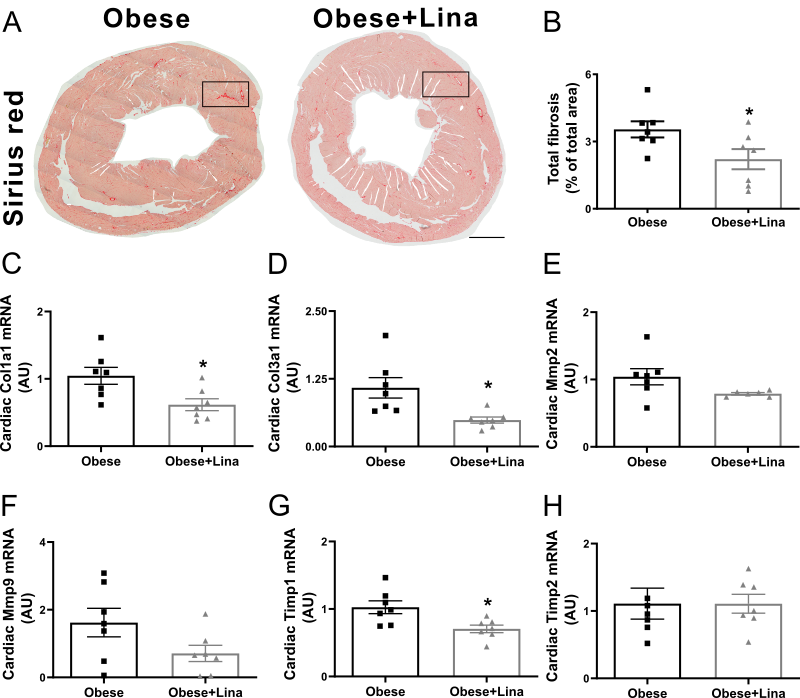


**Figure S4- Linagliptin reduces cardiac fibrosis in obese ZSF1 rats.** A) Representative images of cardiac fibrosis (red) of Sirius red-stained sections in 20-weeks-old linagliptin- (Obese+Lina) and placebo-treated obese (Obese) ZSF1 rats (n=7 per group). Scale bar = 750 µm. The framed area represents the location for the zoomed images presented in Figure 2C. Cardiac total fibrosis (B) and gene expression levels of *Col1a1* (C), *Col3a1* (D), *Mmp2* (E), *Mmp9* (F), *Timp1* (G), and *Timp2* (H) in linagliptin- (Obese+Lina) and placebo-treated obese (Obese) ZSF1 rats at 20 weeks (both =7). Col, collagen; Mmp, matrix metalloproteinase; Timp, tissue inhibitor of matrix metalloproteinase. Data are expressed as mean ± SEM. All graphs were analysed using a two-tailed unpaired Student t-test, except H was analysed by a Mann-Whitney U test with *P<0.05.


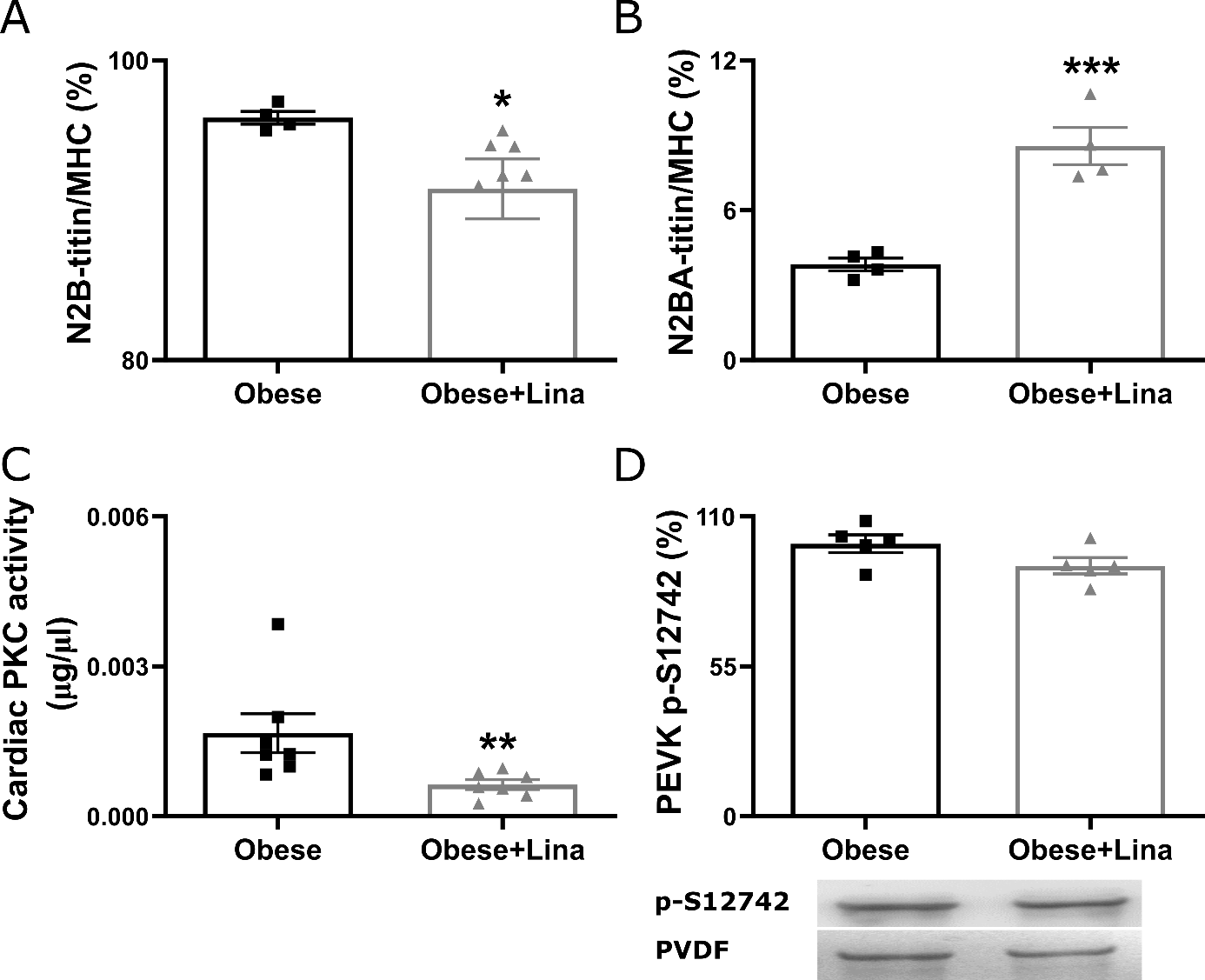


**Figure S5-** **Linagliptin affects titin isoform without changing PKC-site specific titin phosphorylation in obese ZSF1 rats.** Cardiac N2B (A) and N2BA (B) titin isoform/myosin heavy chain (MHC) ratio in 20-weeks-old linagliptin- (Obese+Lina) and placebo-treated obese (Obese) ZSF1 rats (n=4-7/per group). Cardiac PKC activity (C) and PKC-mediated PEVK S12742 phosphorylation (D) in 20-weeks-old linagliptin- (Obese+Lina) and placebo-treated obese (Obese) ZSF1 rats (n=5-7/per group). MHC, myosin heavy chain; PKC, protein kinase C. Data are expressed as mean ± SEM. Graph A, B, and D were analysed using a two-tailed unpaired Student t-test, while graph C was analysed by a Mann-Whitney U test with *P<0.05, **P<0.01, and ***P<0.001.

**
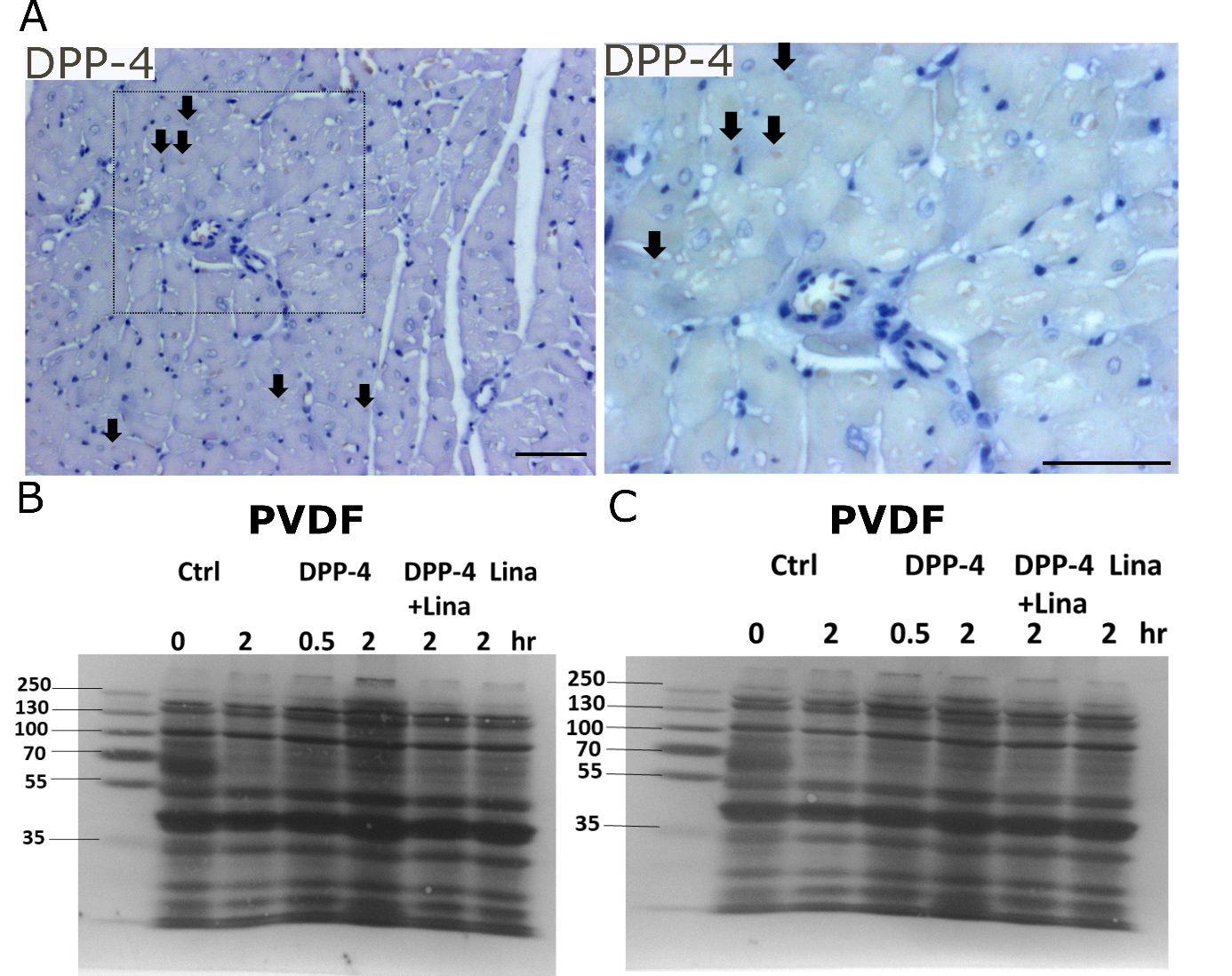
 Figure S6-** **Cardiac DPP-4 expression and loading blots for N2B and PEVK regions of human cardiomyocytes**. A) DPP-4 staining (brown) in cardiac sections of obese ZSF1 rats (Scale bar = 50 µm). The framed area in the right image represents the location for the zoomed images presented on the left. Black arrows indicate the expression of DPP-4 in cardiomyocytes. PDVF loading control membranes for the full specific I-band regions N2Bus (S4043;B) and PEVK (S12884;C) of human cardiomyocytes exposed to control PBS/DMSO for 0 or 2 hours (lane 1 and 2, respectively), 300 ng/ml DPP-4 for 0.5 and 2 hours (lane 3 and 4, respectively), 300 ng/ml DPP-4 and 100 nmol/L linagliptin for 2 hours (lane 5), or 100 nmol/L linagliptin alone for 2 hours (lane 6) (n=3 per condition). Ctrl, control; DPP-4, dipeptidyl peptidase-4.

# **4. Full unedited gels**


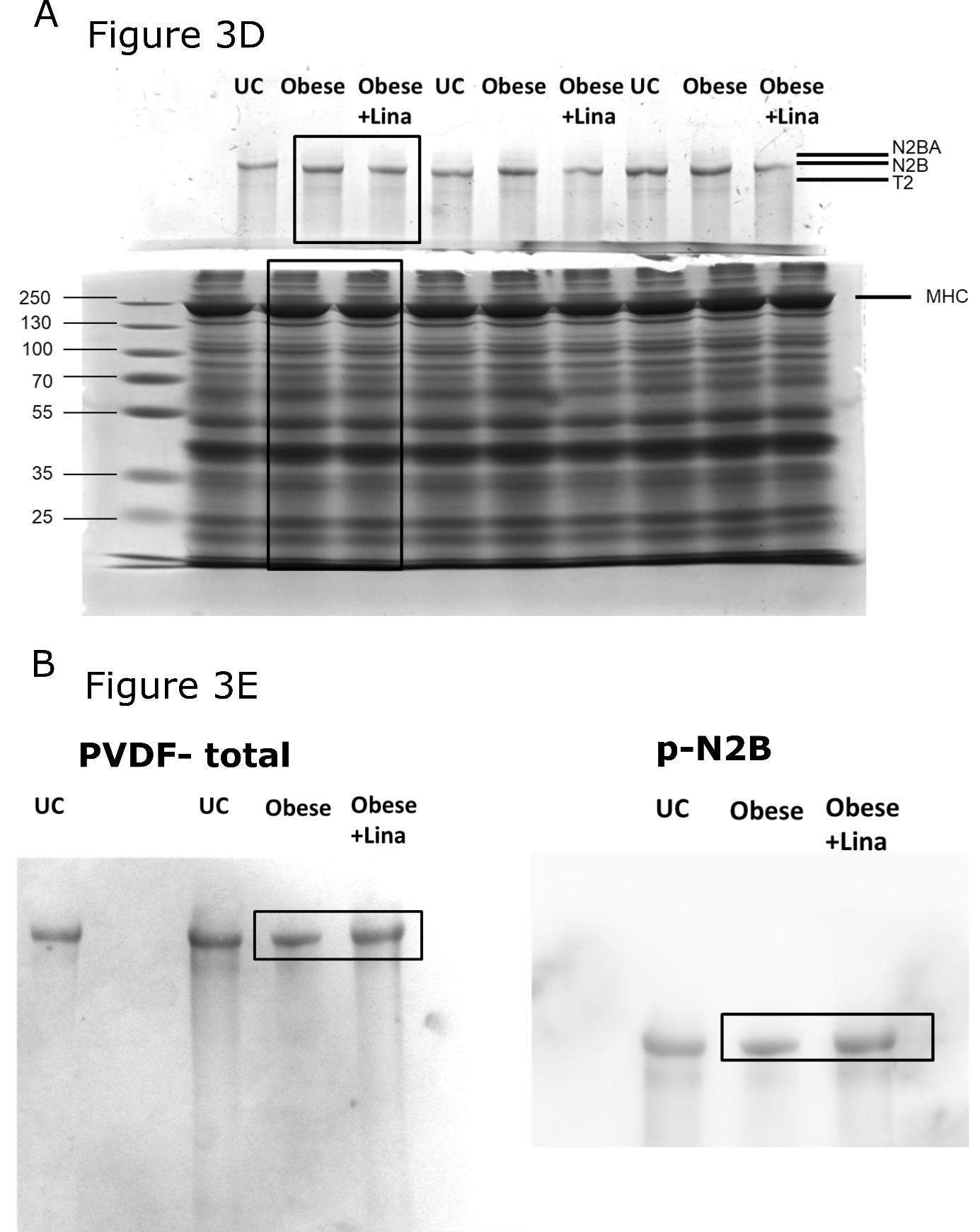


**Full unedited gel Figure 1- Full unedited gels with molecular weights for Figure 3.** A) Full unedited gel for Coomassie blue-stained agarose-strengthened 1.8% SDS-PAGE gel combined with a 10% SDS-PAGE gel for N2B and N2BA isoform detection in unrelated condition (UC), placebo- (Obese) and linagliptin-treated obese (Obese+Lina) ZSF1 rats. Titin-2 (T2) represents a known degradation product of titin. N2BA and N2B isoform presence was normalized for myosin heavy chain (MHC). B) Full unedited Coomassie blue-stained PVDF membranes for full N2B and phosphorylation of N2B in unrelated condition (UC), placebo- (Obese) and linagliptin-treated obese (Obese+Lina) ZSF1 rats. The framed lanes of the unedited gel correspond to the cropped gels in Figure 3.


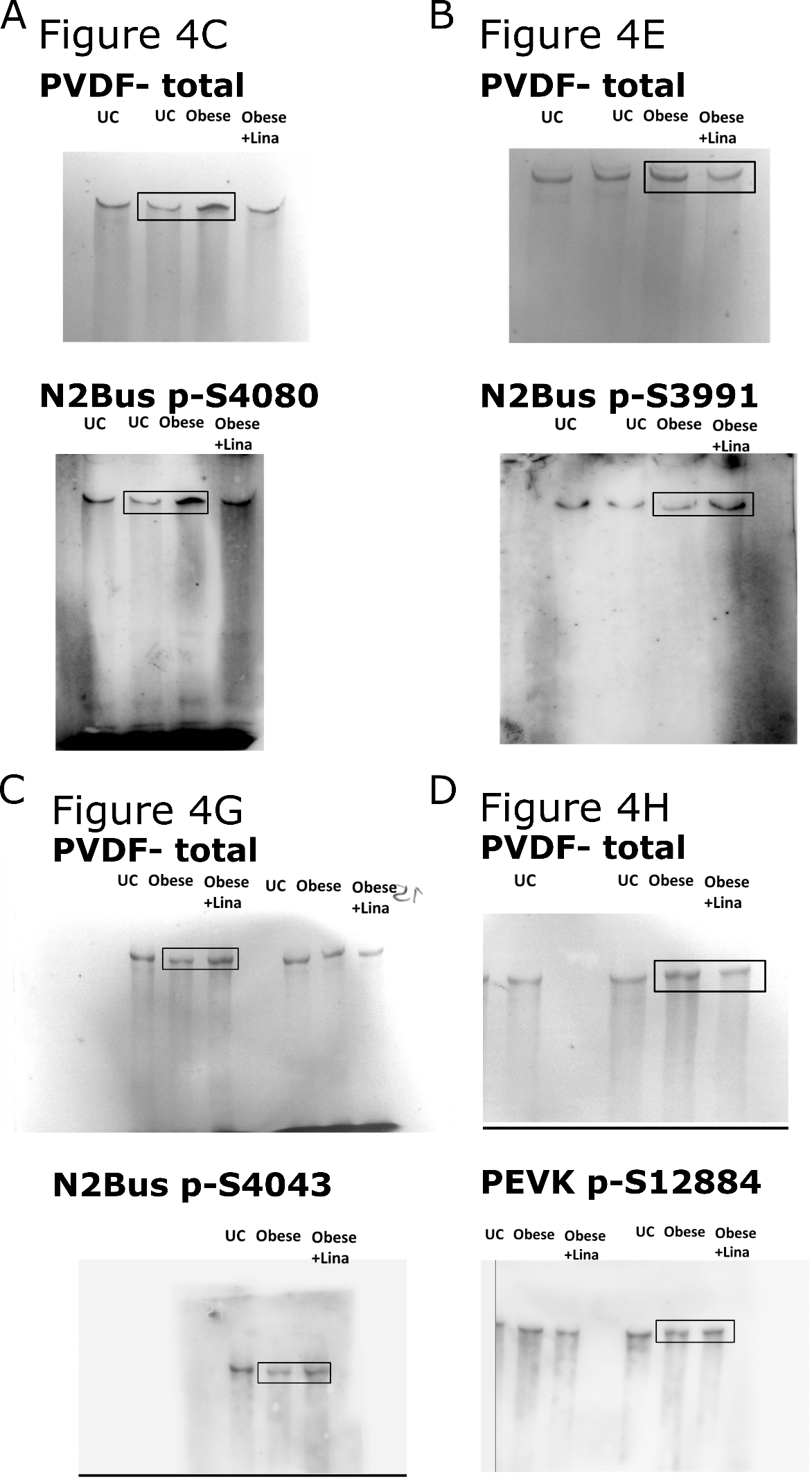


**Full unedited gel Figure 2- Full unedited gels with molecular weights for Figure 4.** Full unedited Coomassie blue-stained PVDF membranes for full and phosphorylated N2Bus S4080 (A), N2Bus S3991 (B), N2Bus 4043 (C), and PEVK S12884 (D) in unrelated condition (UC), placebo- (Obese) and linagliptin-treated obese (Obese+Lina) ZSF1 rats. The framed lanes of the unedited gel correspond to the cropped gels in Figure 4.


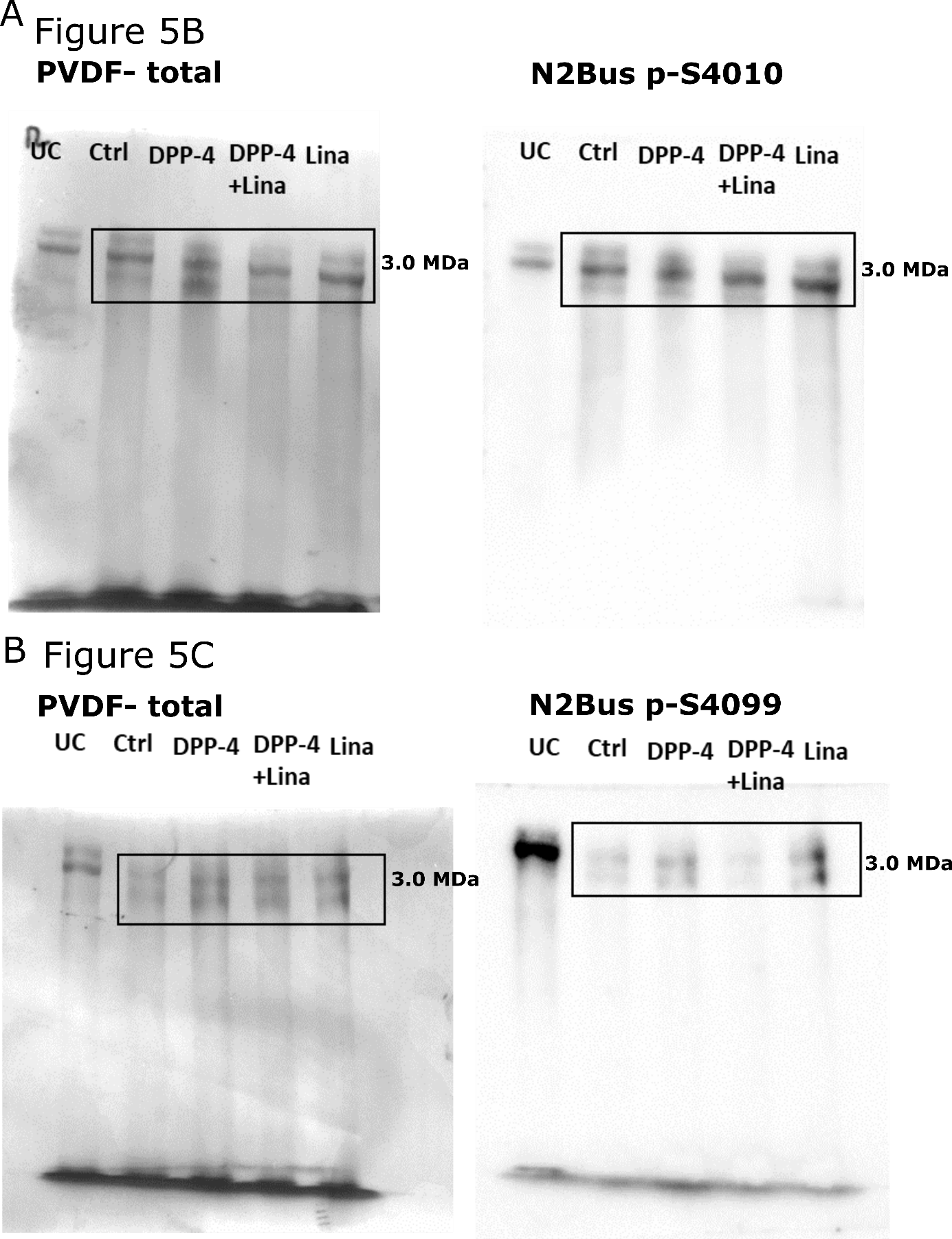


**Full unedited gel Figure 3- Full unedited gels with molecular weights for Figure 5.** Full unedited Coomassie blue-stained PVDF membranes for full and phosphorylated N2B S4010 (A) and N2Bus S4099 (B) in human cardiomyocytes treated *in vitro* with unrelated condition (UC), control (Ctrl), DPP-4, DPP-4 and linagliptin (DPP-4+Lina) and linagliptin (Lina). The framed lanes of the unedited gel correspond to the cropped gels in Figure 5B-C.

**
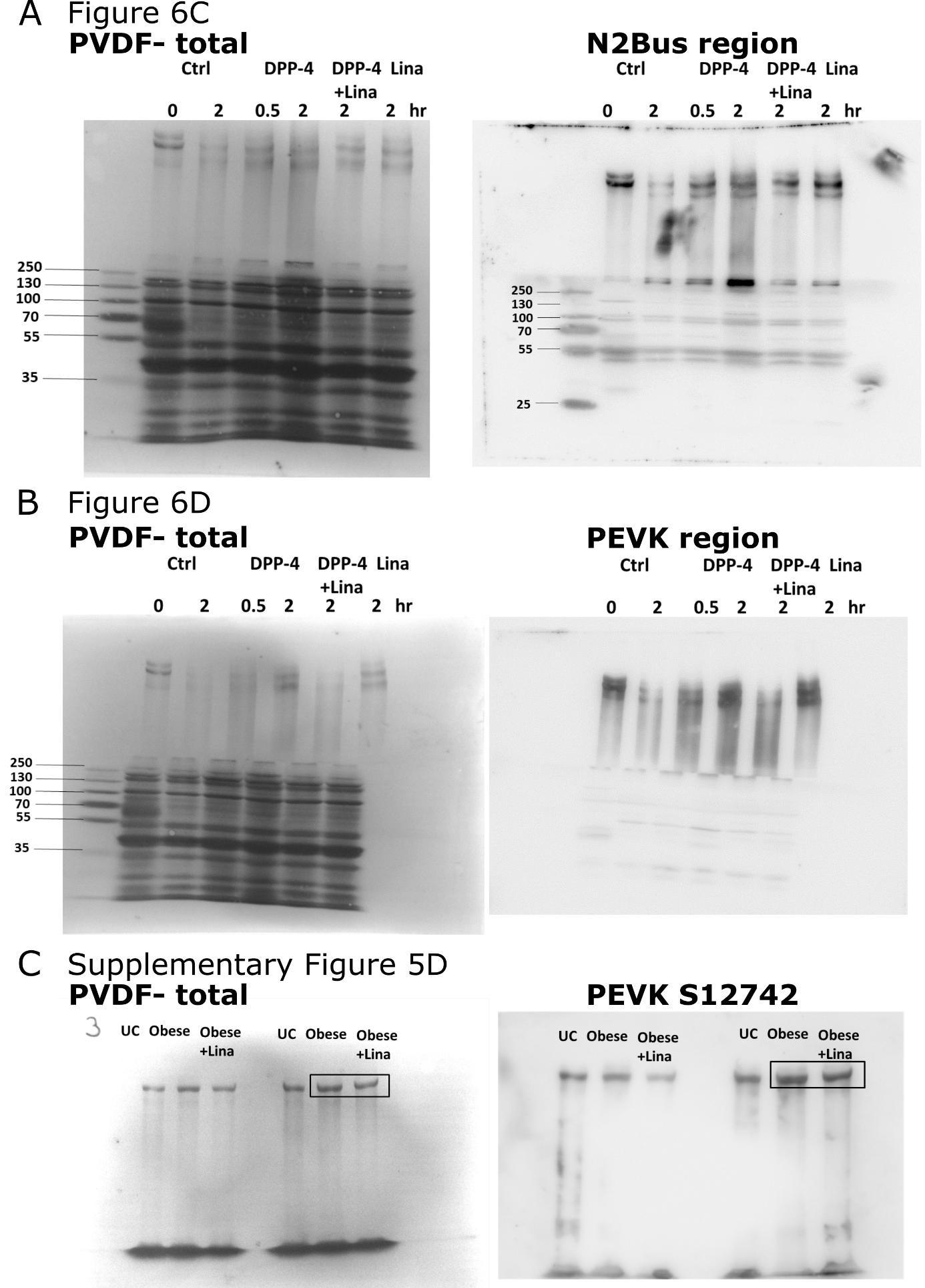
**

**Full unedited gel Figure 4- Full unedited gels with molecular weights for titin cleavage and PVEK S12742 phosphorylation.** Full unedited Coomassie blue-stained PVDF membranes for full N2Bus (A; S346) and PEVK (B;S170) and loading controls of human cardiomyocytes exposed to control PBS/DMSO (Ctrl) for 0 or 2 hours (lane 1 and 2, respectively), 300 ng/ml DPP-4 for 0.5 and 2 hours (lane 3 and 4, respectively), 300 ng/ml DPP-4 and 100 nmol/L linagliptin for 2 hours (lane 5), or 100 nmol/L linagliptin alone for 2 hours (lane 6). The framed lanes of the unedited gel correspond to the cropped gels in Figure 6. Full unedited Coomassie blue-stained PVDF membranes for full PEVK S12742 (C) and its phosphorylation levels in unrelated condition (UC), placebo- (Obese) and linagliptin-treated (Obese+Lina) obese ZSF1 rats. Framed lanes of the unedited gel correspond to the cropped gels in Supplementary Figure 5D.

# **5. References**

1. Dietrich N, Kolibabka M, Busch S, Bugert P, Kaiser U, Lin J, Fleming T, Morcos M, Klein T, Schlotterer A, Hammes HP. The DPP4 Inhibitor Linagliptin Protects from Experimental Diabetic Retinopathy. *PLoS One* 2016;**11**:e0167853.

2. Thomas L, Eckhardt M, Langkopf E, Tadayyon M, Himmelsbach F, Mark M. (R)-8-(3-amino-piperidin-1-yl)-7-but-2-ynyl-3-methyl-1-(4-methyl-quinazolin-2-ylm ethyl)-3,7-dihydro-purine-2,6-dione (BI 1356), a novel xanthine-based dipeptidyl peptidase 4 inhibitor, has a superior potency and longer duration of action compared with other dipeptidyl peptidase-4 inhibitors. *J Pharmacol Exp Ther* 2008;**325**:175-182.

3. Granzier HL, Irving TC. Passive tension in cardiac muscle: contribution of collagen, titin, microtubules, and intermediate filaments. *Biophys J* 1995;**68**:1027-1044.

4. Hamdani N, Franssen C, Lourenco A, Falcao-Pires I, Fontoura D, Leite S, Plettig L, Lopez B, Ottenheijm CA, Becher PM, Gonzalez A, Tschope C, Diez J, Linke WA, Leite-Moreira AF, Paulus WJ. Myocardial titin hypophosphorylation importantly contributes to heart failure with preserved ejection fraction in a rat metabolic risk model. *Circ Heart Fail* 2013;**6**:1239-1249.

5. Hamdani N, Bishu KG, von Frieling-Salewsky M, Redfield MM, Linke WA. Deranged myofilament phosphorylation and function in experimental heart failure with preserved ejection fraction. *Cardiovasc Res* 2013;**97**:464-471.

6. Krysiak J, Unger A, Beckendorf L, Hamdani N, von Frieling-Salewsky M, Redfield MM, Dos Remedios CG, Sheikh F, Gergs U, Boknik P, Linke WA. Protein phosphatase 5 regulates titin phosphorylation and function at a sarcomere-associated mechanosensor complex in cardiomyocytes. *Nat Commun* 2018;**9**:262.

7. Waddingham MT, Sonobe T, Tsuchimochi H, Edgley AJ, Sukumaran V, Chen YC, Hansra SS, Schwenke DO, Umetani K, Aoyama K, Yagi N, Kelly DJ, Gaderi S, Herwig M, Kolijn D, Mugge A, Paulus WJ, Ogo T, Shirai M, Hamdani N, Pearson JT. Diastolic dysfunction is initiated by cardiomyocyte impairment ahead of endothelial dysfunction due to increased oxidative stress and inflammation in an experimental prediabetes model. *J Mol Cell Cardiol* 2019;**137**:119-131.

8. Greaser ML, Berri M, Warren CM, Mozdziak PE. Species variations in cDNA sequence and exon splicing patterns in the extensible I-band region of cardiac titin: relation to passive tension. *J Muscle Res Cell Motil* 2002;**23**:473-482.
